# Contrasted Patterns of Molecular Evolution in Dominant and Recessive Self-Incompatibility Haplotypes in *Arabidopsis*

**DOI:** 10.1371/journal.pgen.1002495

**Published:** 2012-03-22

**Authors:** Pauline M. Goubet, Hélène Bergès, Arnaud Bellec, Elisa Prat, Nicolas Helmstetter, Sophie Mangenot, Sophie Gallina, Anne-Catherine Holl, Isabelle Fobis-Loisy, Xavier Vekemans, Vincent Castric

**Affiliations:** 1 Laboratoire GEPV, CNRS FRE 3268, Univ Lille 1 – Univ Lille Nord de France, Cité Scientifique, Villeneuve d'Ascq, France; 2 Centre National des Ressources Génomiques Végétales, INRA UPR 1258, Castanet-Tolosan, France; 3 Genoscope, Commissariat à l'Energie Atomique (CEA), Direction des Sciences du Vivant, Institut de Génomique, Genoscope, Evry, France; 4 Reproduction et Développement des Plantes, Institut Fédératif de Recherche 128, Centre National de la Recherche Scientifique, Institut National de la Recherche Agronomique, Université Claude Bernard Lyon I, Ecole Normale Supérieure de Lyon, Lyon, France; University of Georgia, United States of America

## Abstract

Self-incompatibility has been considered by geneticists a model system for reproductive biology and balancing selection, but our understanding of the genetic basis and evolution of this molecular lock-and-key system has remained limited by the extreme level of sequence divergence among haplotypes, resulting in a lack of appropriate genomic sequences. In this study, we report and analyze the full sequence of eleven distinct haplotypes of the self-incompatibility locus (S-locus) in two closely related *Arabidopsis* species, obtained from individual BAC libraries. We use this extensive dataset to highlight sharply contrasted patterns of molecular evolution of each of the two genes controlling self-incompatibility themselves, as well as of the genomic region surrounding them. We find strong collinearity of the flanking regions among haplotypes on each side of the S-locus together with high levels of sequence similarity. In contrast, the S-locus region itself shows spectacularly deep gene genealogies, high variability in size and gene organization, as well as complete absence of sequence similarity in intergenic sequences and striking accumulation of transposable elements. Of particular interest, we demonstrate that dominant and recessive S-haplotypes experience sharply contrasted patterns of molecular evolution. Indeed, dominant haplotypes exhibit larger size and a much higher density of transposable elements, being matched only by that in the centromere. Overall, these properties highlight that the S-locus presents many striking similarities with other regions involved in the determination of mating-types, such as sex chromosomes in animals or in plants, or the mating-type locus in fungi and green algae.

## Author Summary

Self-incompatibility is a common genetic system preventing selfing through recognition and rejection of self-pollen in hermaphroditic flowering plants. In the Brassicaceae family, this system is controlled by a single genomic region, called the S-locus, where many distinct specificities segregate in natural populations. In this study, we obtained genomic sequences comprising the S-locus in two closely related Brassicaceae species, *Arabidopsis lyrata* and *A. halleri*, and analyzed their diversity and patterns of molecular evolution. We report compelling evidence that the S-locus presents many similar properties with other genomic regions involved in the determination of mating-types in mammals, insects, plants, or fungi. In particular, in spite of their diversity, these genomic regions all show absence of similarity in intergenic sequences, large depth of genealogies, highly divergent organization, and accumulation of transposable elements. Moreover, some of these features were found to vary according to dominance of the S-locus specificities, suggesting that dominance/recessivity interactions are key drivers of the evolution of this genomic region.

## Introduction

Sexual reproduction entails the combination of genetic material from different individuals to produce offspring. Yet in many species mating is not entirely random, being only possible between individuals with either distinct sexes or distinct mating-types [1]. Sexes or mating-types are typically determined by very distinctive genomic tracts known as sex chromosomes in animals [2], [3] and plants [4], [5], sex-determining loci in honeybees [6], mating-type loci in green algae [7], [8] and fungi [9]–[12] or self-incompatibility (SI) loci in plants [1]. In spite of the wide diversity of organisms and types of molecular and genetic systems involved, these genomic regions typically share several common features. In particular, the genes that directly determine the sexes or the mating-types are often tightly linked, sometimes with a large genomic region containing many genes, in which recombination is suppressed. Such regions can include most of a chromosome (*e.g.* the male-determining region of mammalian Y chromosomes). Recombination suppression in these genomic regions is typically accompanied by a variety of degeneration signatures [13], [10], [2], [14] such as low efficacy of natural selection, low gene density and accumulation of repeated DNA such as transposable elements (TEs).

At present, a comprehensive understanding of the forces driving evolution of these genomic regions is still missing [15]. In particular, two sets of issues remain unanswered. First, the process by which recombination is suppressed and the shape of the transition between recombining and non-recombining regions is not known. In sex chromosomes of mammals and those of the plant *Silene latifolia*, the level of X-Y divergence increases with increasing distance from the boundary with the recombining (pseudo-autosomal) region. Recombination suppression is therefore thought to have occurred in successive and discrete steps [3], [14], [16]–[20], possibly involving large chromosomal inversions. Second, the factors determining the size of the non-recombining region remain poorly understood. In mammals, the size of the Y chromosome is 37% that of the X [3], [14], while in *Silene latifolia* it is 150% that of the X [5].

Homomorphic self-incompatibility (SI) is a highly relevant genetic system to address these issues. SI functions to prevent self-fertilization in hermaphroditic plants [21]. While relatively widespread (being present in at least 94 flowering plant families [22]), homomorphic SI has been described at the molecular level in only a handful of taxa (reviewed in [23], [24]). The genetics of SI involves a single genomic region or a small number of regions. All of the few incompatibility loci that have been characterized at the molecular level contain at least two genes, one expressed in pistils and the other in anthers for sporophytic SI; in gametophytic SI systems, the pollen-S gene is expressed in pollen, and there are sometimes multiple genes [25]. These genes encode proteins that physically interact in a haplotype-specific manner, ultimately allowing normal cross-pollen germination and/or growth when proteins are produced by haplotypes carrying different specificities, but preventing it when pollen and pistils express cognate specificities, in particular avoiding self-fertilization.

Evolutionary properties of the genes controlling SI have been studied in several taxa, including the Brassicaceae, Solanaceae and Papaveraceae species [26], [27]. In accordance with negative frequency-dependent selection theory [28], these genes show remarkable evolutionary features. First, the S-locus typically has very high haplotype diversity, with up to >100 distinct specificities in natural populations within species (see [29] for a review). Second, because they are maintained within species for extended periods of time, these haplotypes show high nucleotide divergence among specificities within species [30] and trans-specific polymorphism between closely related species [31]. Third, to maintain specific recognition, the pollen and pistil genes are expected to be in strong linkage disequilibrium and hence to constitute co-adapted haplotypic combinations [32]. Indeed, recombination between the two component genes would disrupt specific recognition, leading to self-compatible haplotypes [33], [34]. Several studies in different SI systems confirmed that recombination among haplotypes in the S-locus is highly infrequent [35], [33], [36], [34], [37], [30], and consequently that pollen and pistil genes are expected to follow the same evolutionary history. Fourth, in species whose SI system is sporophytic [21], complex dominance relationships have been described among S-haplotypes controlling both pollen and pistil phenotypes [38]. Sporophytic SI has been described at the molecular level in a single family, the Brassicaceae. In both *Brassica* and *Arabidopsis*, the dominance relationships among haplotypes are partly related to their phylogenetic distance, with roughly four different classes in *A. lyrata*, corresponding to four phylogenetic groups [39] and two dominance classes in *Brassica* corresponding to two phylogenetic groups [40], [41]. In line with theoretical expectations [42], [43], dominant and recessive S-haplotypes appear to experience contrasted evolutionary dynamics [30]. In particular, recessive haplotypes generally occur at higher frequency and may form homozygotes. Since molecular polymorphism has been reported among gene copies within a given S-allele [30], homozygote combinations may allow recombination between these highly similar genes copies.

Because of linkage to the targets of negative frequency-dependent selection, the surrounding genomic region is also expected to show deeper coalescence than the genomic background, and hence high sequence divergence among haplotypes [44]. The physical extent of this genomic region is potentially large, in inverse proportion to the extent of local recombination restriction within the S-locus. Analysis of the S-locus in different species belonging to different SI systems confirmed that this genomic region is indeed highly heteromorphic in terms of sequence similarity among haplotypes [45]–[49]. However detailed analyses of the patterns of molecular evolution in the S-locus region are lacking because full sequences of the region are available for just a handful of haplotypes and for a few taxa belonging to different SI systems. In the best documented SI system, that of the Brassicaceae, twelve S-haplotypes have been sequenced in the cultivated species of the *Brassica* genus [50]–[53], [46], [54], [55]. However, many of these sequences lack the flanking regions, hence preventing comparative analysis. In addition, three haplotypes of the S-locus were sequenced in *A. thaliana*, one of which is a recombinant haplotype between two of the three main haplogroups currently segregating in the species [56], [57], [49]. However, although the breakdown of SI is arguably recent in *A. thaliana* [58], the three available sequences encode non-functional haplotypes and may have decayed substantially, especially in light of the rapid genomic changes that occurred since the split with *A. lyrata* [59]. Only five haplotypes from natural populations have been sequenced in Brassicaceae with functional SI, all from *A. lyrata* [60]–[62]. Additionally, two haplotypes with truncated *SCR* sequence, consequently carrying non-functional specificities, were also reported and sequenced in this species [62].

Here, we obtained full sequences for a sample of 11 S-haplotypes from natural populations of *A. halleri* and *A. lyrata*, distributed across the four phylogenetic classes described in these species. We first used these data to determine accurately the boundaries of the non-recombining S-locus region and evaluated its extent, by studying the breakdown of sequence similarity and changes in inter-haplotype phylogenetic patterns at the interface between the flanking regions and the S-locus. We then investigated patterns of variation among haplotypes in the genomic distance between *SCR* and *SRK*, in their relative orientation and in the occurrence of additional ORFs or pseudogenes. We also compared the complement of transposable elements across haplotypes and asked whether the different evolutionary processes acting on dominant and recessive haplotypes had left different molecular signatures. Finally, we took advantage of the complete haplotypic combinations of the two component genes *SCR* and *SRK* in *A. lyrata* and *A. halleri* to investigate their pattern of co-divergence in natural populations.

## Results

The genomic sequences of seven *A. halleri* and four *A. lyrata* S-locus haplotypes were obtained through sequencing of bacterial artificial chromosome (BAC) clones extracted from 9 individual genomic libraries. Libraries were screened with probes from the two genes immediately flanking the S-locus region (*U-box* and *ARK3*). Positive clones were checked using BAC-end sequencing and further validated by PCR targeted on *SRK* sequences using haplotype-specific primers [63]. Full BAC sequences were then obtained using 454 pyrosequencing technology. Because of the large sequence divergence among haplotypes, individual sequencing reads were assembled *de novo*, resulting in two to nine large contigs for each clone, with an average clone size of 98 kb and mean coverage of 57×. Attempts to increase coverage did not eliminate the gaps, suggesting that they may contain repetitive sequences. To reject the hypothesis of non-functional *SCR* or *SRK* genes, we used long-range PCR to validate the proposed assemblies when assembly gaps occurred within *SCR* or *SRK* introns (*AhSRK15*, *AlSRK01*, *AlSCR39* and *AhSCR03*). All these PCR resulted in successful amplifications and the different exons of SCR or SRK were thus confirmed to be consecutive. Detailed characteristics of the BAC clone sequences are reported in Table S1.

### Recombination suppression and the boundaries of the S-locus

**Figure 1:**
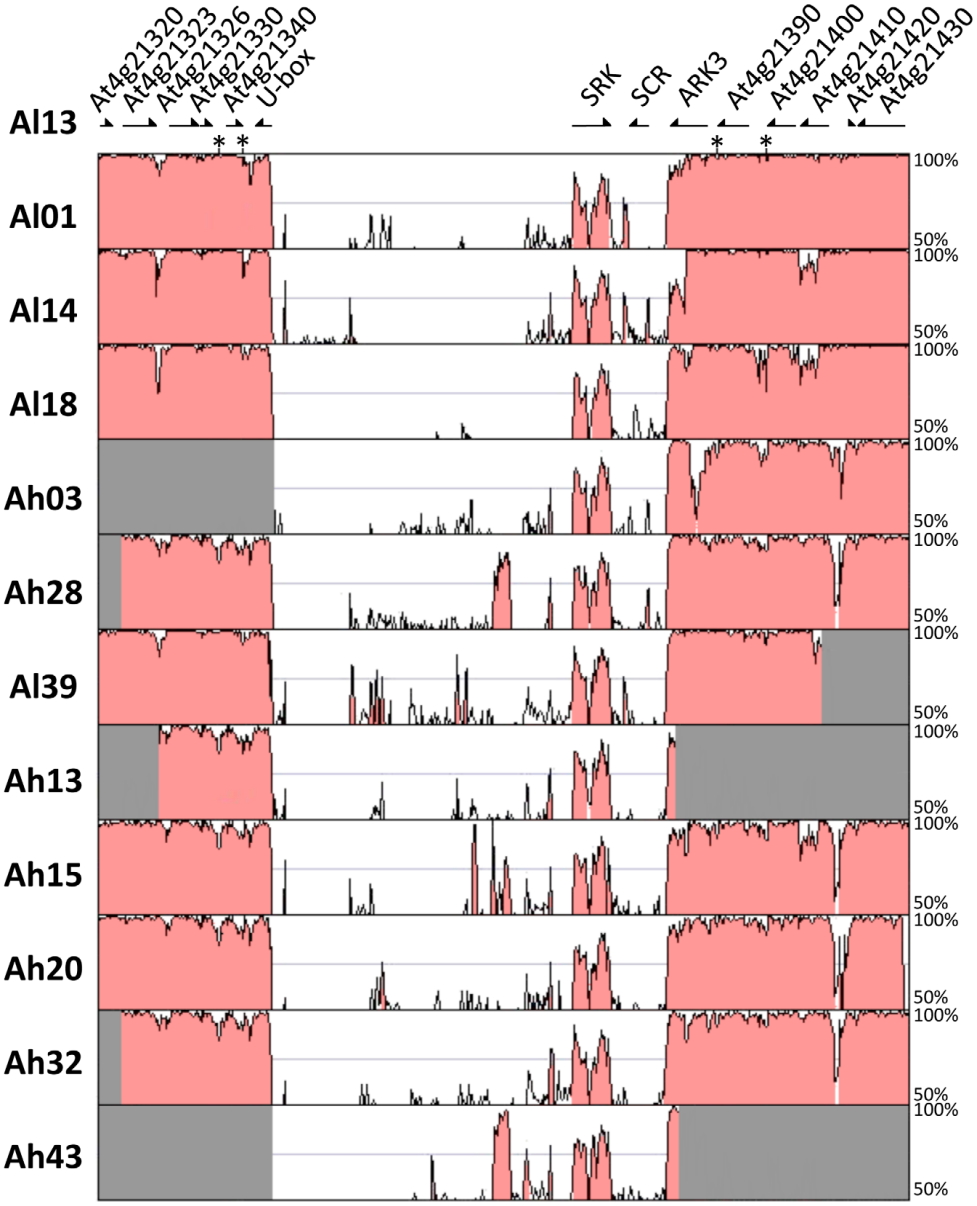
Sequence conservation in the S-locus region between *Al13* (the reference *A. lyrata* genome) and each of the other haplotypes. Note that the figure is not to scale except for the reference sequence. Portions of sequences not available for some haplotypes were colored in gray. For clarity, transposable elements outside of the S-locus in *Al13* were extracted from the sequence, and their locations are indicated by an asterisk.

To determine the precise location of the boundaries of the non-recombining S-locus region, we compared sequences from twelve S-locus haplotypes (additionally including the reference haplotype *Al13* from the *A. lyrata* full genome sequence [59]) using the VISTA software [64], looking for a transition in the levels of sequence similarity among haplotypes. As shown in Figure 1 and Figure S1, the sequence conservation among different haplotypes is fairly high in flanking regions on both sides of the S-locus, but plummets sharply between about 300 bp upstream of the start codon of the *U-box* gene on one side and near the stop codon of *ARK3* on the other side. Hence, we define the S-locus as this region of very low similarity lying between these two breakpoints. Synteny is remarkably well conserved outside the S-locus region, except for the presence or absence of some transposable elements in intergenic regions (which were removed from the reference sequence in Figure 1 for clarity). High sequence similarity among haplotypes and high collinearity of flanking genes in the region outside of the S-locus suggest that recombination among haplotypes does occur outside the region delimited by these breakpoints. Additional evidence comes from the observation that elevated diversity, as expected for neutral sites linked to sites under balancing selection [44], is mostly apparent for the two immediately flanking genes (the *U-box* and *ARK3*), while levels of synonymous nucleotide diversity are comparable with that of the genomic background (≈2%, [65], [66]) for genes located further away on the chromosome (Figure S2), as previously reported [37], [65]. In contrast, within the S-locus, sequence similarity is almost completely lacking, the only notable exceptions being the seven exons of *SRK* and some transposable elements of the same family. Interestingly, a pseudogenized partial duplicate of the *ARK3* gene (from the end of the first exon to the end of the gene) is found within the S-locus in three different haplotypes: *Al01*, *Ah15* and *Ah43*. These partial duplicates of *ARK3* within the S-locus region could be responsible for the observation by Hagenblad *et al.* [67] of the occurrence of a pseudogenized paralog of *ARK3* in some haplotypes, including one carrying allele *Al01* at *SRK*. A similar partial duplicate sequence of *ARK3* was found in the S-locus region of the recombinant *C24* haplotype of *A. thaliana*, and it was hypothesized that this motif acted as the recombination breakpoint between the two common haplotypes *A* and *C* [57]. Interestingly, the duplicated *ARK3* sequences in *Al01*, *Ah15* and *Ah43* are more similar to *ARK3* gene copies present in haplotypes other than their own (Figure S3). Assuming that this second copy initially originated through gene duplication from the same chromosome, this observation implies that inter-haplotype recombination does occur at the genomic position of this gene, and hence supports our conclusion that *ARK3* indeed lies outside the non-recombining region. Moreover, while the partial duplicates of *ARK3* in *Ah15* and *Ah43* are closely related, that of *Al01* is not phylogenetically close, suggesting at least two independent duplication events.

### The S-locus has low gene density and shows important structural rearrangements

**Figure 2:**
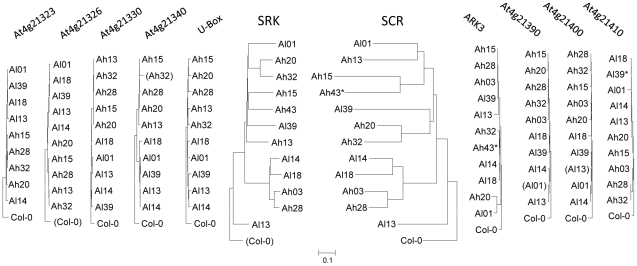
Gene phylogenies in and around the S-locus region. Phylogenies were obtained by the Minimum Evolution method, and are based on coding sequences, with the *A. thaliana* reference sequence (Col-0) as an outgroup. Asterisks indicate partial sequences, and brackets non functional sequences. The inversion in the *SCR* coding sequence of Col-0 was de-inverted (*i.e.* restored to its original functional configuration in *A. halleri*) according to Tsuchimatsu *et al.* [70]. Separate phylogenies for each gene are available in Figure S4.

Annotation of the S-locus region revealed only the two incompatibility genes, *SCR* and *SRK*, plus TEs (see below). A single copy of *SCR* and of *SRK* was found in each haplotype, whereas a previous study [60] described two copies of *SCR* in one haplotype from *A. lyrata* (*Al20*). Multiple gene copies are therefore the exception rather than the rule in the S-locus of *Arabidopsis*. Sequencing of the 206.7 Mb *A. lyrata* genome predicted 32,670 genes [59], *i.e.* approximately 0.16 genes per kb. With only two genes in about 60 kb, the S-locus appears to have very low gene density (*ca.* 4.8 times lower than the genomic background). Striking differences in the timescales of gene genealogies for the S-locus genes *SCR* and *SRK* as compared to the flanking genes were observed (Figure 2), with much deeper genealogies for *SCR* and *SRK*, as expected for genes under strong frequency-dependent selection [68]. Moreover, the gene genealogies of *SCR* and *SRK* (Figure 2) were found to be more congruent than expected by chance (I_cong_ = 1.53; P-value = 0.0014 [69]). Specifically, the phylogenetic classes defined based on *SRK* sequences [39] (class I: *Al01*; class II: *Ah03*, *Ah28*, *Al18* and *Al14*; class III: *Al13*; class IV: all other haplotypes) are conserved in the *SCR* tree.

In contrast, the phylogenetic relationships among haplotypes were strikingly different for the flanking genes (Figure S4), as reported for haplotypes of the *U-box* and the *ARK3* genes in *A. thaliana* [70]. Indeed, in our dataset gene genealogies of the flanking genes tend to cluster according to species overall, rather than to S-locus phylogenetic classes. This observation further supports the conclusion that the non-recombining region is confined to the S-locus and is determined by the two breakpoints identified based on sequence similarity.

**Figure 3:**
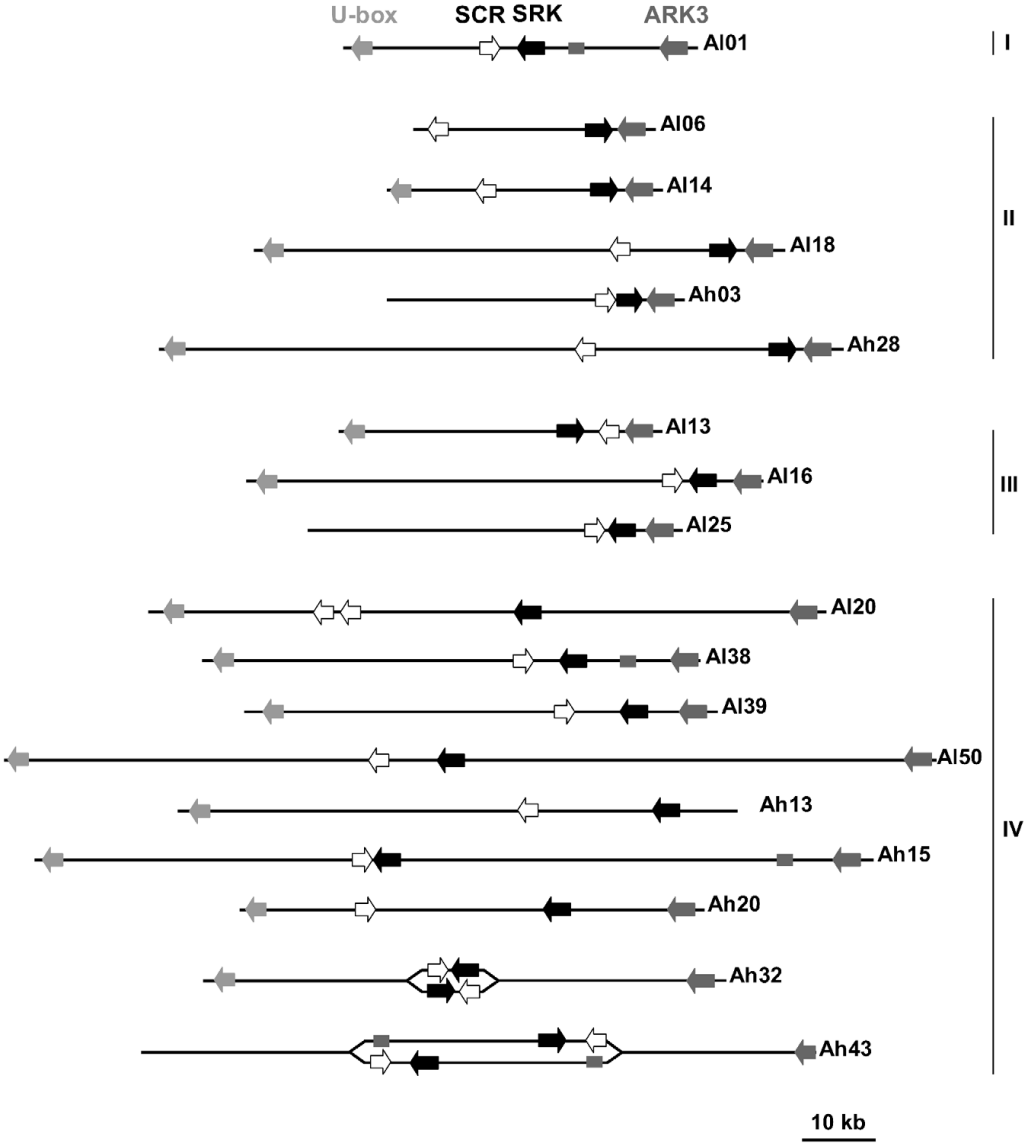
Structural variation within the S-locus. The direction of *SCR*, *SRK* and the two flanking genes is shown taking into account their approximate distances. Both possibilities are depicted when the orientation of genes remains unknown due to unoriented contigs. The presence of a pseudo-*ARK3* sequence is represented by a dark gray rectangle. Organization of haplotypes *Al20*, *Al06*, *Al25*, *Al16*, *Al38* and *Al50* are based on Kusaba *et al.* [60], Boggs *et al.* [61] and Guo *et al.* [62].

The S-locus region is variable in size across haplotypes, spanning from 31 kb (haplotype *Al14*) to 110 kb (haplotype *Ah15*) with an average size of 62 kb. Given that BAC sequences do not cover the totality of the S-locus from haplotypes *Ah03*, *Ah13* and *Ah43*, these estimates are lower bounds. Also, several libraries that we constructed could not be exploited because no single clone showed both flanking genes used for screening, suggesting that the S-locus haplotypes they contain may have been larger than the average 100 kb typical of the BAC clones in our libraries. With an average size of 74 kb, haplotypes from *SRK* phylogenetic class IV are generally larger than haplotypes from classes I to III, showing an average size of 50 kb (Table 1). Figure 3 summarizes the gene organization within the S-locus and includes data from Kusaba *et al.* [60], Boggs *et al.* [61] and Guo *et al.* [62]. Globally, we found that gene organization within the S-locus is highly variable with regard to gene order (*SRK* located either on the *ARK3* or the *U-box* side as compared to *SCR*, although the latter order was only found in a single haplotype, *Al13*), relative orientation of *SCR* and *SRK* (tail-to-tail, head-to-head or in the same direction), and distance separating them (from less than 1 kb to about 26 kb; Table 1). These patterns also vary among haplotypes within each of the *SRK* phylogenetic classes, with the exception of class II haplotypes showing mostly *SCR* and *SRK* oriented tail-to-tail and a location of *SRK* consistently very close to the flanking gene *ARK3* in head-to-head orientation. Strikingly, these class II haplotypes were already reported to show common features that distinguish them from other phylogenetic classes [71], [39]. We found here that the strong sequence similarity previously noted in the kinase domain of these haplotypes [71] is extended to the whole intergenic region (about 900 bp in length) between *SRK* and *ARK3* (Figure S5), in contrast to comparisons with other classes of haplotypes or between classes (Figure S1). As suggested by [39], these class II haplotypes could have originated by a gene conversion event implying unlinked members of the *SRK* gene family. Interestingly, this same intergenic region is also conserved between class II haplotypes and haplotypes *Ah15* and *Ah43*, two of the three haplotypes carrying a pseudogeneized duplicated copy of *ARK3*. This observation strongly suggests that the duplication involved a recombination event between these haplotypes and a class II haplotype. Interestingly, while [62] suggested that haplotypes *Al38* and *Al50* lack the second exon of the *SCR* gene, we were able to detect the second exon upon closer examination applying the same approach than in our own data, suggesting that these haplotypes are indeed functional. In addition, while previous studies failed to detect a kinase domain for *AlSRK01* [30], our genomic approach confirmed that all *SRK* sequences we observed contained a full-length kinase domain.

**Table 1:** Description of the different haplotypes.

Haplotype	Phylogenetic class	Size of the S-locus	*SCR* - *SRK* distance
*Al01*	I	42 614	2 906
*Al14*	II	30 909	8 671^a^
*Al18*	II	65 495	12 227
*Ah03*	II	34 512	742
*Ah28*	II	87 805	25 748
*Al13*	III	37 013	1 752
*Al39*	IV	55 787	6 601
*Ah13*	IV	73 401	17 028
*Ah15*	IV	109 864	618
*Ah20*	IV	56 764	3 636^a^
*Ah32*	IV	52 987	1 974
*Ah43*	IV	93 791	4 147

a Because of the uncertainty on the orientation of some contigs, the indicated distance is the minimum distance between *SCR* and *SRK*.

### Invasion by transposable elements and the effect of dominance

**Figure 4:**
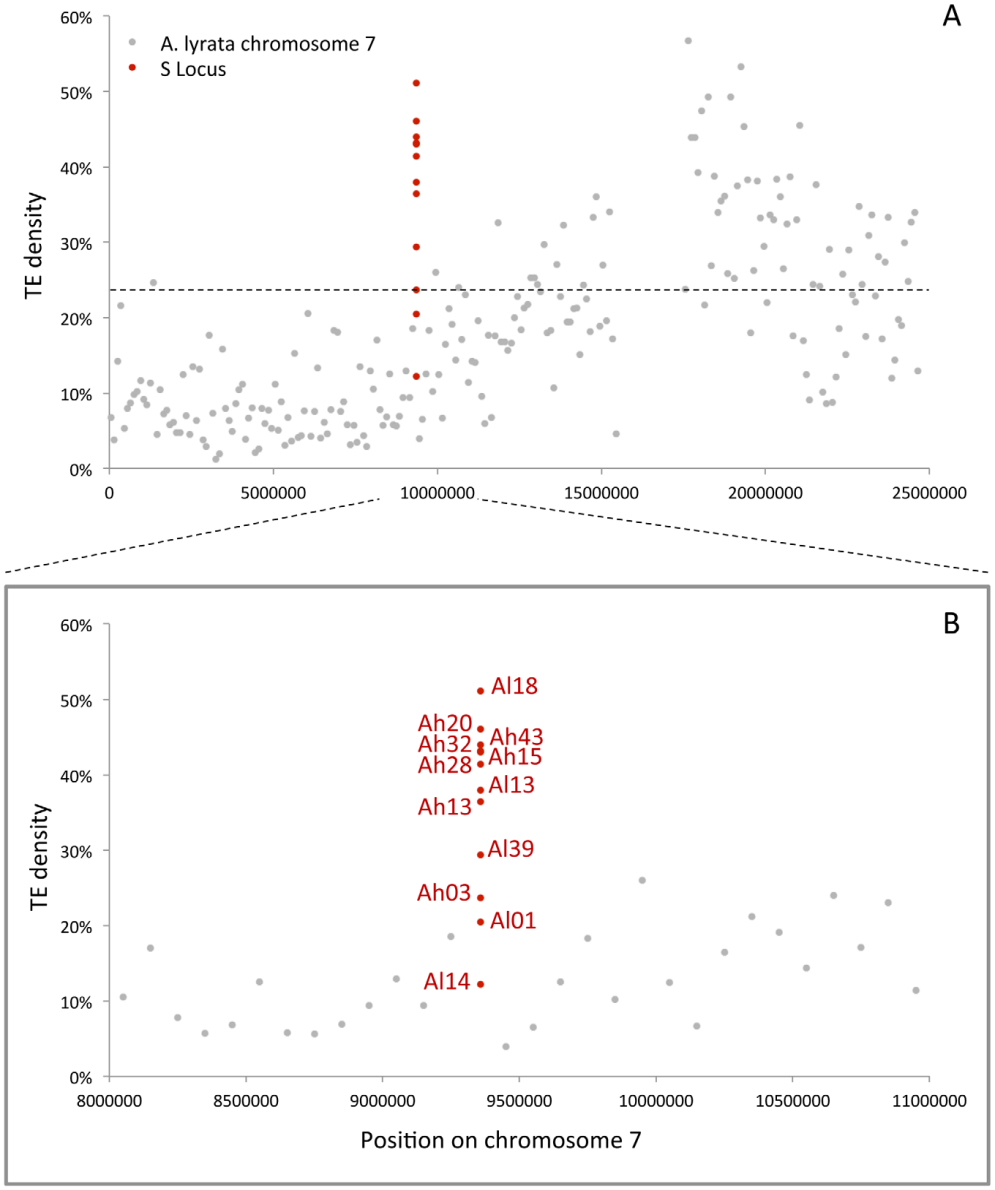
TE density along *A. lyrata* chromosome 7, comparison with the S-locus data, zoom on the 3 Mbp region around the S-locus. A. TE density along *A. lyrata* chromosome 7, and comparison with the S-locus data. Transposable elements contents were calculated using CENSOR [72] for non overlapping windows of 100 kb. B. Zoom on the 3 Mbp region around the S-locus. The dashed line represents a 95% confidence interval on the TE densities of this 3 Mbp genomic region.

Transposable elements annotation with the CENSOR [72] and PLOTREP [73] programs revealed a strong density and diversified complements of TEs in the S-locus, with a representation of most families known in the *A. thaliana* genome (detailed annotation and a complete list of TEs for each haplotype are shown in Figure S6 and Table S2). In order to determine whether these observations are uncommon in the genomic background, we also used CENSOR [72] to estimate TE density along the *A. lyrata* genome divided in non-overlapping windows of 100 kb. Variation of TE density along chromosome 7 confirmed that the TE density of the S-locus sharply departs from its chromosomal background, being matched only by the centromeric region (Figure 4, and Figure S7 for the other chromosomes). This difference is not due to an invasion by a single class of TEs, since the quantitative difference in density was observed for most TE families (Figure S8).

**Figure 5:**
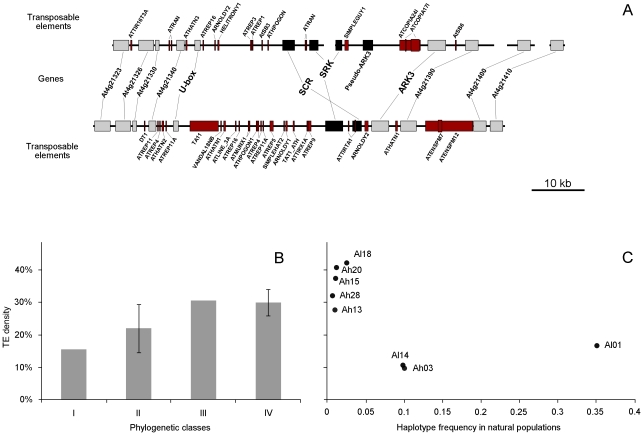
Comparative annotation of genes and transposable elements, mean TE density, and TE density according to frequency. A. Comparative annotation of genes and transposable elements for a recessive haplotype, *Al01*, and a dominant one, *Al13*. The S-locus genes are represented by black rectangles, and other genes by light gray ones. Transposable elements, annotated using CENSOR [72] and PLOTREP [73], are indicated in red. B. Mean TE density in the different phylogenetic classes. TE density corresponds to the percentage of a sequence which is covered by transposable elements. Standard deviation is indicated for classes II and IV, in which multiple haplotypes could be analysed. C. TE density of the different haplotypes according to their frequency in natural populations. Haplotype frequencies are based on *SRK* fragments. *A. lyrata* data concern 12 icelandic populations [76] and A. *halleri* data concern 39 european populations (unpublished data).

While most haplotypes have higher TE density than the genomic background, there is striking variability in TE density among haplotypes. Indeed, TE density depends on *SRK* phylogenetic classes, which are themselves associated with dominance with higher density in the more dominant haplotypes (Figure 5A and 5B). Since levels of dominance are in turn expected to correlate with S-haplotype frequency in natural populations [74], [42], [75], we plotted TE density against haplotype frequency, as estimated from S-locus genotype surveys in *A. lyrata* [76] and *A. halleri* (P. Goubet *et al.* unpublished data). We find that variation in TE density is even better captured by haplotype frequencies, with rare haplotypes being more enriched in TEs than more frequent haplotypes (Figure 5C).

## Discussion

Our results confirm that the S-locus in *A. halleri* and *A. lyrata* differs significantly from its genomic background in several respects: gene density is particularly low, gene genealogies are much deeper as compared to the flanking genes, gene order and orientation vary extensively, sequence similarity among haplotypes in intergenic sequences is completely lacking and the density of transposable elements is particularly elevated, being matched only by that in the centromere. Most of these properties are shared with many genetic systems controlling patterns of mating such as sex chromosomes, sex-determining loci or mating-type loci.

### Size of genomic regions involved in mating-type determination

Contrasted patterns of conservation between the S-locus and its flanking regions are in line with two previous investigations comparing three and five haplotypes in *A. thaliana* [49] and *A. lyrata* [62], respectively. Based on a more extensive collection of S-haplotypes, we could precisely map the breakdown of synteny to two narrow regions very close to the 5′ or 3′ ends of the coding regions of the flanking genes *U-box* or *ARK3*, respectively. Note that [37] and [65] reported some level of co-segregation of flanking genes with variation at *SRK* in local *A. lyrata* populations, while our global sample from two related species might have left sufficient time for recombination to uncouple the S-locus region from its genomic background. Using this objective criterion to define the S-locus itself, we find that the S-locus has an average size of 62 kb, ranging from 31 to 110 kb among haplotypes, much larger than the average distance of 7 kb between the two S-locus genes, *SCR* and *SRK*, ranging from 1 kb to 26 kb. An orthologous sporophytic SI system occurs in the genus *Brassica*, although the S-locus is located in a different genomic region than in *Arabidopsis*. Based on the available sequences of four *B. rapa* haplotypes [53], [55] and using a similar criterion to define the S-locus we determined that the S-locus was somewhat smaller than in *Arabidopsis*, ranging from 28 to more than 60 kb. In contrast, the distance separating *SCR* and *SRK* was less variable, ranging from 2 to 11 kb. In *Brassica*, however, the S-locus generally comprises a third gene, *SLG* which is a paralog of *SRK* lacking the kinase domain, and the overall region comprising these three genes ranged from 23 to 43 kb. In *Ipomoea trifida* (Convolvulaceae), which also exhibits sporophytic SI but of a different molecular nature, the S-haplotype-specific divergent region between the only two sequenced haplotypes (*S1* and *S10*) extends over 50 and 34 kb, respectively [77]. In the gametophytic SI system of *Prunus dulcis* and *P. mume* (Rosaceae), the S-locus was estimated as being a divergent genomic region of about 70 kb [78] and 15 to 27 kb [45], respectively. In *Antirrhinum hispanicum* (Plantaginaceae), the distance between the two component genes of haplotype *S_2_* is 9 kb [79]. However, a major difference between the S-locus of the Brassicaceae and that of the Plantaginaceae and Solanaceae is that in the latter the pollen phenotype can be encoded by different members of the gene family to which the male determinant belongs, so that the S-locus comprises more than two genes [25], making this comparison tricky. Overall, in spite of the large diversity of species and molecular mechanisms involved in the different SI systems, the size of S-loci seems to be fairly constant across taxa, ranging from 27 to about 110 kb, with *Arabidopsis* species apparently in the upper part of the range.

Beyond the comparison with S-loci of other plants, the size of the S-locus can also be compared with that of the mating-type loci in fungi or green algae. In the basidiomycete *Cryptococcus neoformans*, sex determination is controlled by a locus including genes encoding a pheromone and its receptor. Haplotypes of this mating-type locus, α and a, represent a genomic region of approximately 105 to 130 kb [80], hence slightly larger than the S-locus in *A. halleri* and *A. lyrata*. In another basidiomycete, *Ustilago hordei*, the mating-type locus consists of a single region comprising two complexes, a and b, between which recombination is suppressed. The distance between these two complexes was estimated to be 500 kb and 413 kb in the *MAT-1* and *MAT-2* strains, respectively [9]. In the ascomycete *Neurospora tetrasperma*, the non-recombining region comprising the mating-type locus covers 78.4% of the chromosome length, *i.e.* 6.9 Mbp [11]. In green algae, the mating-type locus of the unicellular *Chlamydomonas reinhardtii* consists of a highly rearranged 200-kb region [81] while that of the multicellular *Volvox carteri* is about 500% larger and contains many ORFs. Interestingly, *C. reinhardtii* is an isogamous species with two morphologically indistinguishable mating-types [7] while *V. carteri* shows morphological differentiation between the mating-types, suggesting the general conclusion that genomic regions involved in mating-type systems that are not associated with morphological differences between mates may span smaller genomic regions. In other words, the accumulation of genes with a role in expression of the morphological differences between mating-types [8] may contribute to some extent to the variation in size of the mating-type locus, in addition to transposable elements and non coding DNA accumulating in these regions. Because in homomorphic SI the mating-types are not associated with morphological differences, the S-loci may retain a smaller size.

### Structural rearrangements, yet shared evolutionary history, between SCR and SRK

Only six sequences of *SCR* were previously described in *Arabidopsis* because of the difficulty of finding conserved regions to perform PCR amplifications [60], [61], [82], [70], [62]. Our important sequencing effort of the S-locus region resulted in the successful identification of full *SCR* sequences in ten new S-haplotypes in *A. halleri* and *A. lyrata* and only the second exon of *SCR* in one haplotype (haplotype *Ah43*, for which we could not obtain the full S-locus sequence), along with their cognate *SRK* partner. These results do not support the hypothesis of existence of non-functional haplotypes carrying only partial SCR sequences, as proposed by Guo *et al.* [62], as we were able to localize the missing coding sequence for their two putative non-functional haplotypes when applying the ALN [83] software fed with all known *SCR* sequences. Congruence of *SCR* and *SRK* phylogenies reflects the coevolution necessary to maintain the specific SCR-SRK protein-protein recognition, and clearly indicates that recombination between the two SI genes has been precluded. Comparison of phylogenies between *SCR* and the S domain of *SRK* was already investigated by Sato *et al.* [32] for twelve haplotypes in *Brassica oleraceae*. They found that the hypothesis of an identical topology for the two trees was not rejected. Edh *et al.* [84] also compared *SCR* and *SRK* phylogenies in *Brassica rapa*, *Brassica oleraceae* and *Brassica cretica* class II haplotypes, but congruence between topologies could not be clearly demonstrated, perhaps as a consequence of the concerted evolution of the *SLG* and *SRK* genes within haplotypes, or of the more recent history of diversification within the class II lineage. In contrast, in the ascomycete *Neurospora* [85], the non-self recognition system is controlled by two tightly linked genes, *het-c* and *pin-c*. In agreement with our results in the S-locus, congruence was found between topologies of the phylogenies of these two genes, but not with those of the flanking genes. When more *SCR/SRK* sequences become available, it will be interesting to study in more details the co-evolutionary process.

Based on the study of nine haplotypes in *A. thaliana*, *A. lyrata* and *Capsella rubella*, Guo *et al.* [62] proposed that head-to-head orientation of *SCR* and *SRK* was the ancestral orientation in the *Arabidopsis/Capsella* lineage. However, the lack of conserved orientation pattern in our results based on a much larger number of haplotypes suggests that, in spite of the shared evolutionary history of *SCR* and *SRK*, the S-locus has experienced a history of frequent inversions and genomic rearrangements. At this stage, we argue that the ancestral orientation cannot be deduced. However, our results confirm that with a single exception *SCR* always occurs at the *U-box* side and *SRK* at the *ARK3* side. Interestingly, the exception to this rule concerns haplotype *Al13*, which was obtained from an *A. lyrata* strain (MN47) with non-functional SI. This suggests the intriguing possibility that the observed inversion may have been associated with the breakdown of SI in this strain used for sequencing the *A. lyrata* genome. Strong structural variation among haplotypes seems to be a common feature of S-loci [86] and genomic rearrangements, particularly inversions, are known to be frequent in low recombination regions such as in sex chromosomes of mammals [3], [14], [87] and plants [19] or in the mating-type locus of green algae [81]. Evidence suggesting gradual suppression of recombination was found in sex chromosomes, and led to the concept of evolutionary strata [16]–[18], [3], [14], [19], [20]. These strata, composed of genes which stop recombining and therefore start diverging presumably at the same time, could have been caused by large inversions in the non-recombining sex chromosome [16]. As in sex chromosomes, inversions in the S-locus could have contributed to the reduction in recombination among haplotypes. However, no discrete strata of divergence among haplotypes can be identified. Instead, the proportion of sequence similarity changes abruptly to mostly zero within the S-locus region.

### Transposable elements accumulation in sex-determining regions

Our results show that transposable elements are a major component of the S-locus region, as previously noted in other taxa [47], [48], [54]. On a wide scale, their density is higher in most S-haplotypes than in the genomic background. Such accumulation has already been observed in other genomic regions involved in mating-type and gender determination, and is not exclusive to the S-locus. Bachtrog [2] investigated four regions of the neo-sex chromosomes, containing homologous gene pairs, in *Drosophila miranda*. In each case, the neo-Y showed several transposable elements insertions that were absent from the neo-X. Similarly, Marais *et al.* [5] analyzed genetic degeneration of the Y chromosome in *Silene latifolia*, by examining seven sex-linked genes. Comparison of Y-linked genes and their X-linked homologs provided evidence that some of the Y-linked genes showed higher intron sizes, due to the accumulation of transposable elements. In the mating-type locus of the basidiomycete *Ustilago hordei*, sequencing of one of the two haplotypes, *MAT-1*, revealed that this genomic region was particularly rich in both retroelements and repetitive DNA compared to *U. maydis*, in which the a and b complexes are unlinked [88]. Similarly, the chromosome carrying the mating-type locus in the fungus *Microbotryum violaceum* was found to be enriched in transposable elements as compared to autosomal chromosomes [10]. In *A. thaliana*, Wright *et al.* [89] compared the transposable elements accumulation in chromosome arms and in low-recombining regions surrounding the centromeres, *i.e.* centromeres, pericentromeric regions and heterochromatic knobs. These regions of reduced recombination were shown to exhibit greater TE copy numbers than chromosome arms, particularly for Gypsy retrotransposons and EnSpm transposons. Interestingly, our results showed that precisely these two TE families present densities twice higher in the S-locus than in the overall genome of *A. lyrata*, suggesting that the increased TE density noticed in the S-locus is effectively linked to the restricted recombination.

### TE accumulation: Driven by recombination suppression and mutational hazard?

Strikingly, we found that not all haplotypes present the same TE coverage, with dominant S-haplotypes (*SRK* phylogenetic classes III and IV) having higher TE density than those belonging to recessive classes (I and II). Signatures of intragenic recombination have been found in *SRK* only in S-haplotypes belonging to recessive classes I and II [30]. It was suggested that recombination can occur only in individuals carrying two copies of the same functional S-haplotype, which is most probable for recessive haplotypes, because they are predicted to have high frequencies in natural populations [42]. Indeed, in *A. lyrata*, the most recessive haplotype was 12.75 times commoner than the most dominant haplotypes in Icelandic natural populations [90]. Our observation that TE density is inversely related to haplotype population frequency also suggests that recombination plays a role in preventing TE accumulation in the S-locus. In addition, haplotype frequency also influences the effective population size of gene copies within S-haplotypes [68], so that genetic drift will be stronger in low-frequency dominant haplotypes (in agreement with the mutational-hazard model of Lynch and Conery [91]), and this may also affect TE accumulation. Sex chromosomes in mammals also differ in opportunities for recombination and in effective population sizes [91]. Recessive S-haplotypes tend to behave like the X chromosome, and dominant ones are more like the Y chromosome. These differences may be an important source of variation of the size of the S-locus among haplotypes.

## Methods

### Construction of BAC libraries

High Molecular Weight (HMW) DNA was prepared from young leaves of seven *A. halleri* and four *A. lyrata* haplotypes. For each extraction, approximately 20 grams of frozen leaf tissue was ground to powder in liquid nitrogen with a mortar and pestle used to prepare megabase-size DNA embedded in agarose plugs. HMW DNA of the various genotypes was prepared as described by Peterson *et al.* [92] and modified as described in [93]. Embedded HMW DNA was partially digested with *Hind*III (New England Biolabs, Ipswich, Massachusetts), subjected to two size selection steps by pulsed- field electrophoresis, using a BioRad CHEF Mapper system (Bio-Rad Laboratories, Hercules, California), and ligated to pIndigoBAC-5 *Hind*III-Cloning Ready vector (Epicentre Biotecnologies, Madison, Wisconsin). Pulsed-field migration programs, electrophoresis buffer, and ligation desalting conditions were performed according to [94].

To evaluate the average insert size of each library, BAC DNA was isolated from about 384 randomly selected clones in each library, restriction enzyme digested with the rare cutter NotI, and analyzed by Pulsed-Field Gel Electrophoresis (PFGE). All fragments generated by NotI digestion contained the 7.5 kb vector band and various insert fragments. Analysis of the insert sizes from the various BAC libraries showed a mean insert size comprised between 80 kb and 175 kb. Since the haploid genome of *A. lyrata* and *A. halleri* is estimated around 230 Mb and 250 Mb respectively, we picked the number of BAC clones required to obtain a library coverage of 5 genome equivalents.

### Screening the BAC libraries

High-density colony filters were prepared from all the nine BAC libraries constructed using a robotic workstation QPix2 XT (Genetix). BAC clones were spotted in double using a 5×5 or 6×6 pattern onto 22×22 cm Immobilon-Ny+ filters (Millipore Corporate, Billerica, Massachusetts). On each filter, 27 648 to 41 472 unique clones were spotted in duplicate, and clones were grown at 37°C for 17 h. Filters were then processed as follows: (1) denaturation on Whatman paper soaked with a solution of 0.5 M NaOH and 1.5 M NaCl for 4 min at room temperature, and for 10 min at 100°C, (2) neutralization on Whatman paper soaked with 1 M TrisHCl pH 7.4, and 1.5 M NaCl for 10 min, incubation in a solution of 0.25 mg/mL proteinase K (Sigma Aldrich, St. Louis, Missouri) for 45 min at 37°C, baking for 45 min at 80°C, and (3) fixation by UV on a Biolink 254 nm crosslinker (Thermo Fischer Scientific, Waltham, Massachusetts) with an energy of 120,000 µJoules. Radiolabelling of probes and hybridization of the filters were performed as described in [93]. Hybridized filters were imaged with a Storm 860 PhosphorImager (GE Healthcare, Little Chalfont, UK), and analyses were performed using the HDFR software (Incogen, Williamsburg, Virginia). Positive BAC clones detected by hybridization were validated individually by PCR amplification using the primer pairs used for probes synthesis (Table S3), and visualisation of PCR products after agarose gel electrophoresis.

### Sequencing

A total of fourteen BACs covering the S-locus region of 11 S-haplotypes were sequenced in this study (two partially overlapping BACs were needed for haplotypes *Al28*, *Ah15* and *Ah28*) : eleven BACs were sequenced at Genoscope; two BACs (containing haplotypes *Al39* and *Ah43*) were sequenced at CNRGV; and a last one (haplotype *Al14*) was sequenced by Beckman Coulter 485 Genomics.. All clones were sequenced using a 454 multiplexing technology on Titanium sequencer (www.roche.com). De-novo assembly was performed by Newbler (www.roche.com) for each S-haplotype and only contigs representing the extremities of the BACs were organized at this step.

### Sequence finishing

BAC sequences covering the 11 S-haplotypes were obtained in two to nine contigs. Suggestion of orientation was provided with assembly for some sequences, but in most cases, only the first and last contigs were oriented. The relative order and orientation of other contigs were therefore unknown. When exons of *SCR* or *SRK* were in two different contigs (*i.e.* haplotypes *Al01* and *Ah15* for *SRK*, *Ah03* and *Al39* for *SCR*), primers were defined with Primer3 [95] on both contigs. Because of the presence of repeated sequences including transposable elements, long-range (using TaKaRa LA Taq Polymerase) rather than classical PCR were performed in order to confirm the contiguity of the contigs.

### Sequence annotation

Annotation of BAC sequences was performed using two gene structure prediction programs with *Arabidopsis* parameters, FGENESH [96] and GENSCAN [97]. FGENESH has the advantage of being more accurate in detecting *Arabidopsis* genes but GENSCAN is more sensitive. Detected ORFs were blasted using BLASTX [98] and the obtained proteins were then aligned on BAC sequences with SPALN [99] and FGENESH+ [96] softwares. Because of its high nucleotide diversity, *SCR* was rarely detected by these two programs. Known *SCR* proteins were therefore aligned on BAC sequences using the semiglobal alignment procedure implemented on ALN [83], which is more sensitive than SPALN and FGENESH+. The results were then examined by eye in order to find the *SCR* gene and the cysteine residues that characterize this protein. Transposable elements were annotated with CENSOR [72] using the *A. thaliana* repetitive elements [v16.02] database of Repbase Update [100]. The results were then filtered and defragmented with PLOTREP [73], using a minimum coverage of merged fragments of 10%.

### Comparison of sequences and phylogenetic analysis

The full BAC sequences were aligned and compared using the “glocal” alignment procedure [101] implemented in VISTA [64]. This kind of alignment is able to detect rearrangements and inversions in sequences, and is particularly appropriate for divergent regions like the S-locus. Protein sequences of genes were aligned with CLUSTALW [102]. Alignments were then manually adjusted and phylogenetic trees were constructed using MEGA version 5 [103], according to a Minimum Evolution (ME) analysis with the maximum composite likelihood method. The congruence between topologies of *SCR* and *SRK* trees was tested by computing an index of congruence, based on the size of their maximum agreement subtree, and comparing its value to a null-hypothesis distribution obtained by simulation of random trees [69].

### Analysis of the transposable elements content

A PERL script was developed to compare TE density between the twelve S haplotypes and the *A. lyrata* genome. CENSOR [72] was used in local on BAC sequences, excluding the S-locus flanking regions, and on non-overlapping windows of 100 kb along the eight chromosomal sequences of the *A. lyrata* genome version Araly1 (http://genome.jgi-psf.org/Araly1/Araly1.download.html [59]). Densities were thus calculated for each transposable element family in the *A. lyrata* genome and in the S-locus, according to the classification in [104].

## Supporting Information

Figure S1 Sequence conservation at the S-locus boundaries between *Al13* (the reference *A. lyrata* genome) and each of the other haplotypes. Sequences not available for the *U-box* side (*Ah03* and *Ah43*) were not represented. Distance from the homology breakpoint is indicated under each graph.
(PDF)

Figure S2 Synonymous nucleotide diversity (Π_S_) at S-locus flanking genes for *A. halleri* (black) and *A. lyrata* (gray), estimated using DnaSP [105].
(PDF)

Figure S3 Phylogeny of *pseudo-ARK3* sequences, *SRK* and *ARK3*. Phylogeny was constructed using a Minimum Evolution analysis.
(PDF)

Figure S4 Separate phylogenies of the S-locus Region genes. Phylogenies were obtained by the Minimum Evolution method, and are based on protein sequences, with the *A. thaliana* reference sequences (Col-0) as outgroup.
(PDF)

Figure S5 Sequence conservation in the *SRK-ARK3* region between *Ah28* (Class II) and each of the other haplotypes. Distance from homology breakpoint is indicated under the graph.
(PDF)

Figure S6 Annotation of genes and transposable elements for the 12 S-haplotypes. The S-locus genes are represented in black rectangles, with delimitation of their exons. Other genes are depicted in light gray. Transposable elements are shown in dark gray, and their fragmentation is indicated by white gaps.
(PDF)

Figure S7 TE density along *A. lyrata* chromosomes 1 to 6 and chromosome 8. Transposable elements contents were calculated using CENSOR [72] for non overlapping windows of 100 kb.
(PDF)

Figure S8 Comparative density in different families of transposable elements for the entire genome of *A. lyrata*, and the S-locus of *A. lyrata* and *A. halleri*. Transposable elements classification refers to Wicker et al. [104].
(PDF)

Table S1 Description of the different clones. Two clones were necessary to cover the entire S-locus for three haplotypes : *Al18*, *Ah28* and *Ah15*.
(DOC)

Table S2 List of the transposable elements detected in the BAC sequences.
(DOC)

Table S3 Primers pairs used to validate BAC clones by PCR amplification. These primers were defined to amplify *SRK* (primer Sh04), *U-box* (primer B80) and *ARK3* (primer ARK3) genes.
(DOC)
