# Supplementary figures and images for: Contrasted Patterns of Molecular Evolution in Dominant and Recessive Self-Incompatibility Haplotypes in Arabidopsis
Source: PLoS Genet. 2012 Mar 22;8(3):e1002495. doi: 10.1371/journal.pgen.1002495 (PMC3310759; doi:10.1371/journal.pgen.1002495)

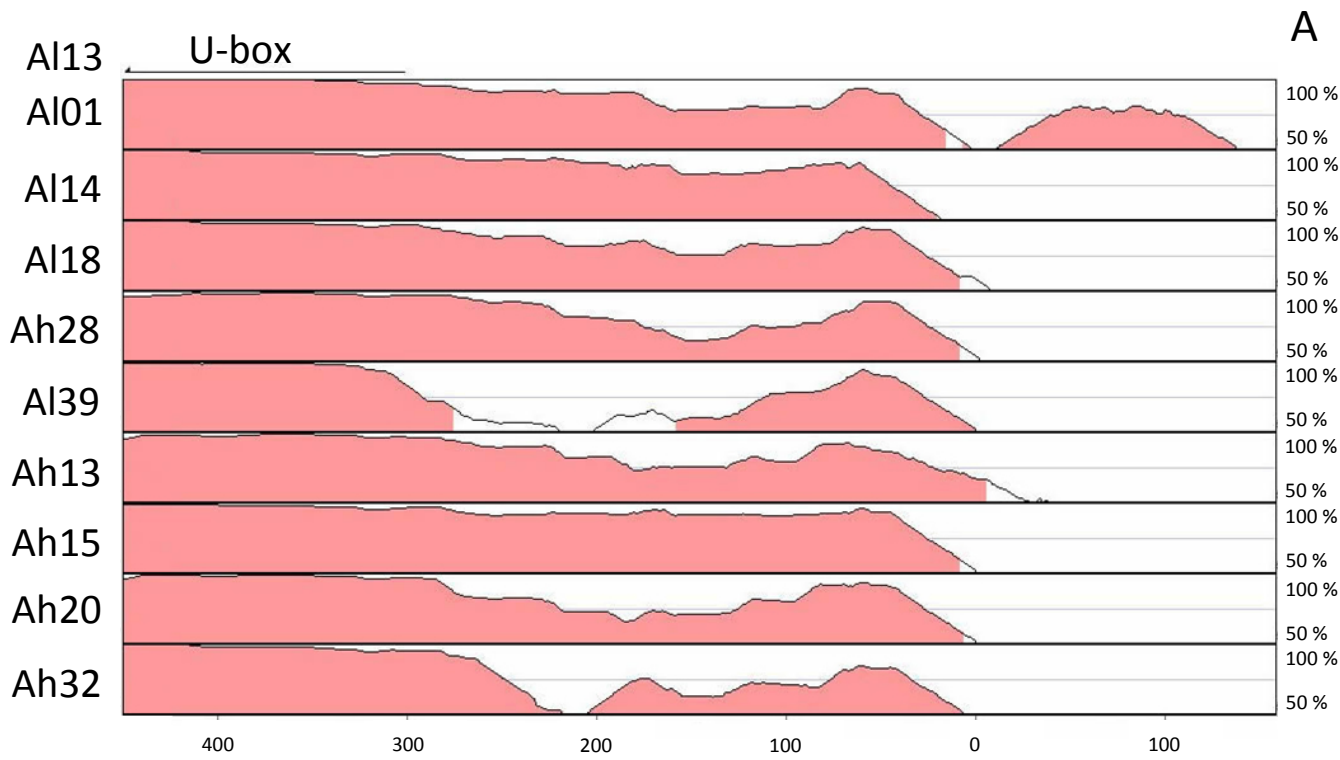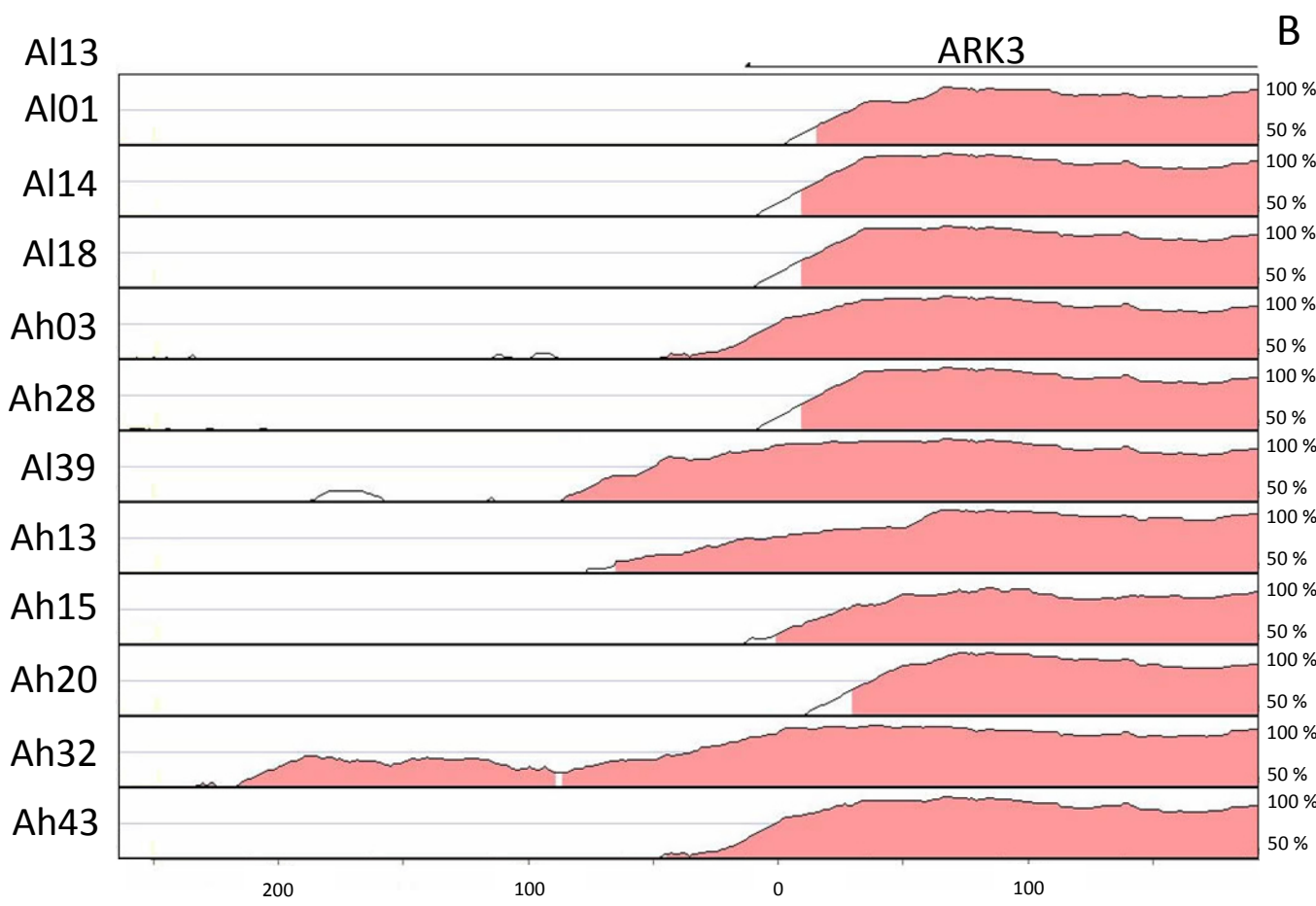

Supplement: Figure S1 — Sequence conservation at the S-locus boundaries between Al13 (the reference A. lyrata genome) and each of the other haplotypes. Sequences not available for the U-box side (Ah03 and Ah43) were not represented. Distance from the homology breakpoint is indicated under each graph. (PDF) [file pgen.1002495.s001.pdf]

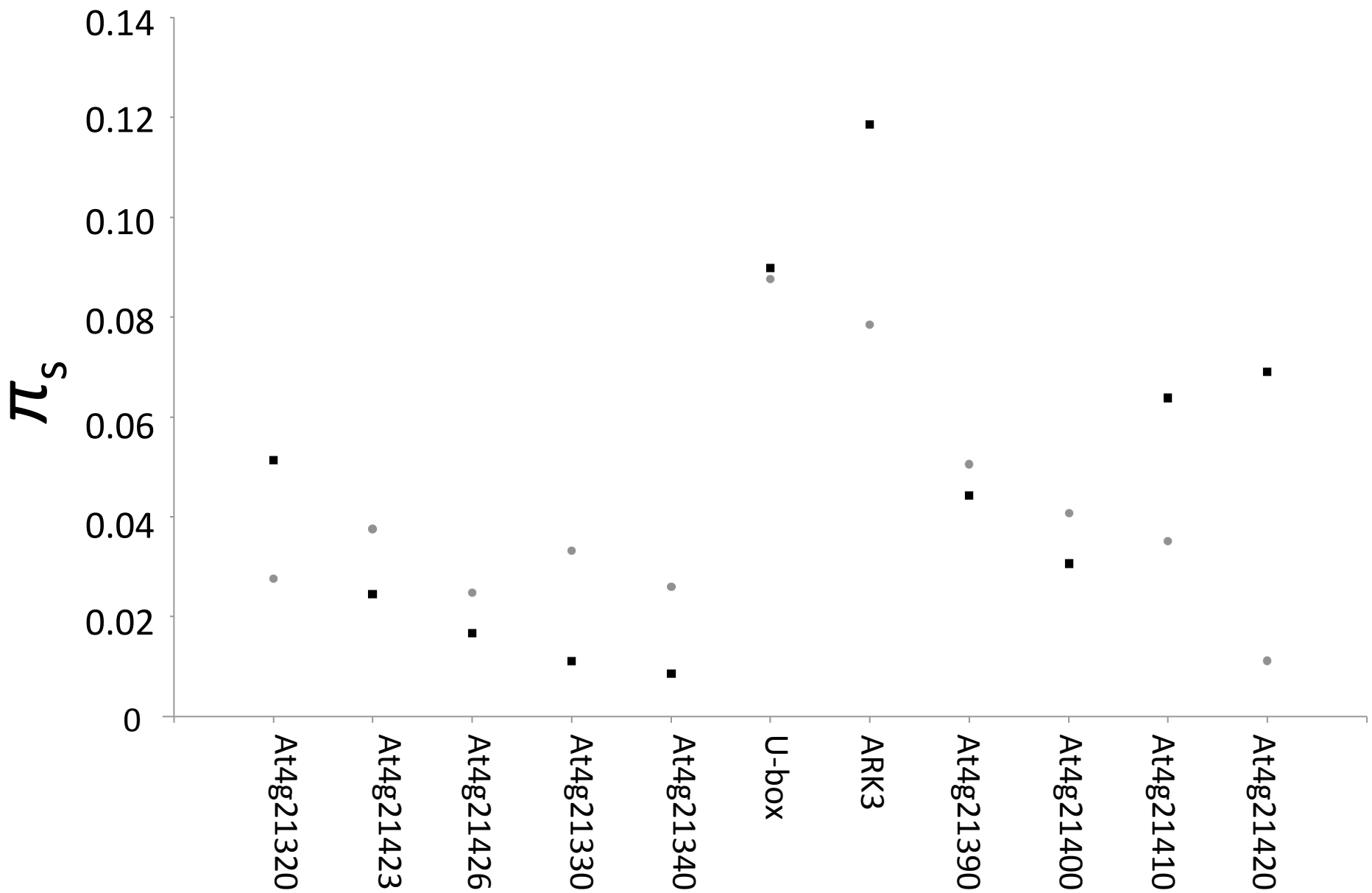

Supplement: Figure S2 — Synonymous nucleotide diversity (ΠS) at S-locus flanking genes for A. halleri (black) and A. lyrata (gray), estimated using DnaSP [105]. (PDF) [file pgen.1002495.s002.pdf]

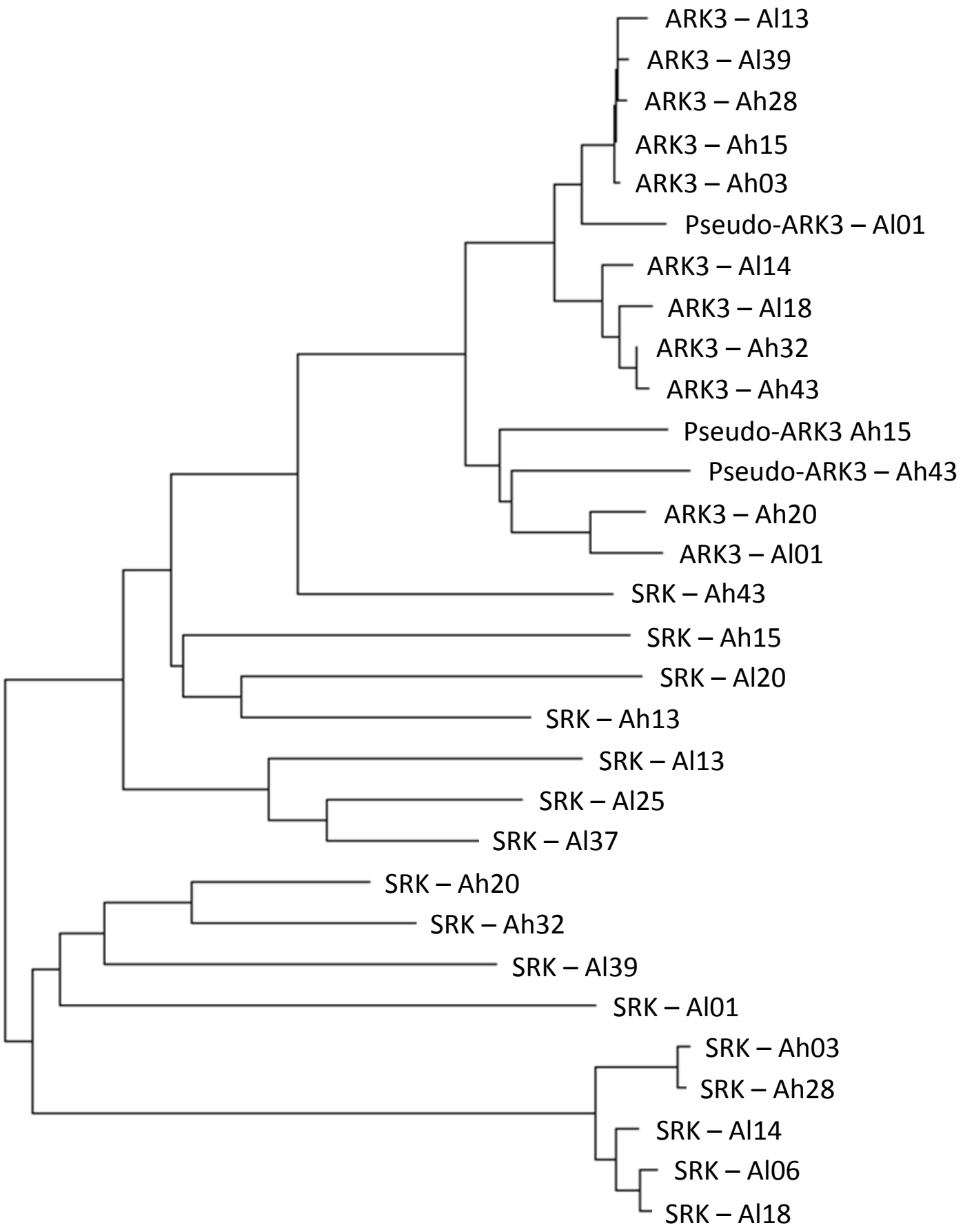

0.02

Supplement: Figure S3 — Phylogeny of pseudo-ARK3 sequences, SRK and ARK3. Phylogeny was constructed using a Minimum Evolution analysis. (PDF) [file pgen.1002495.s003.pdf]

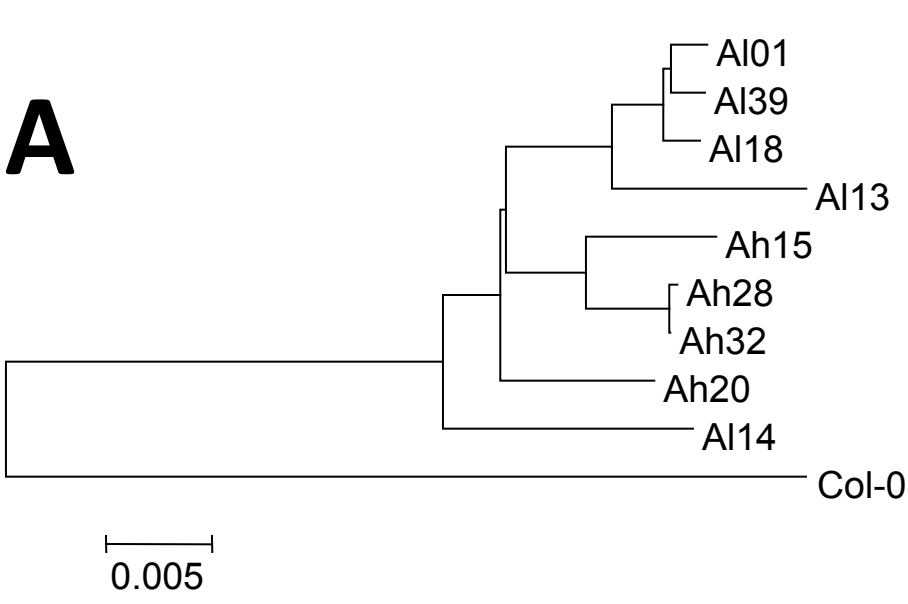

***At4g21323***

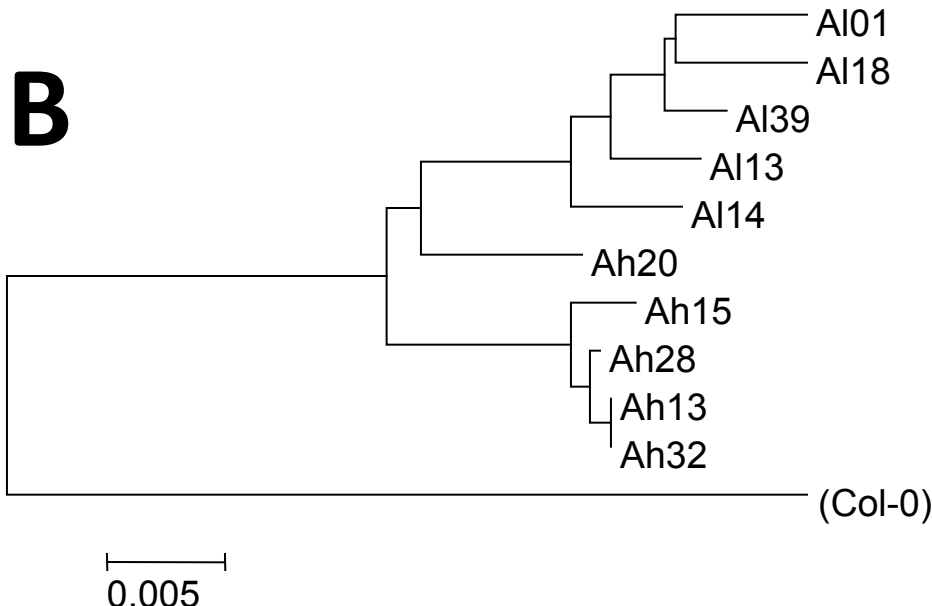

***At4g21326***

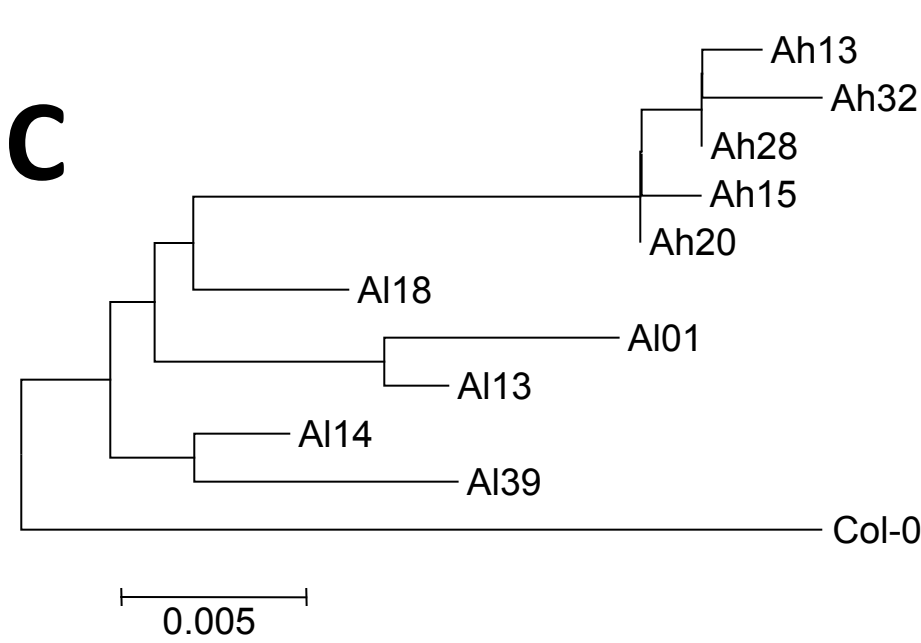

***At4g21330***

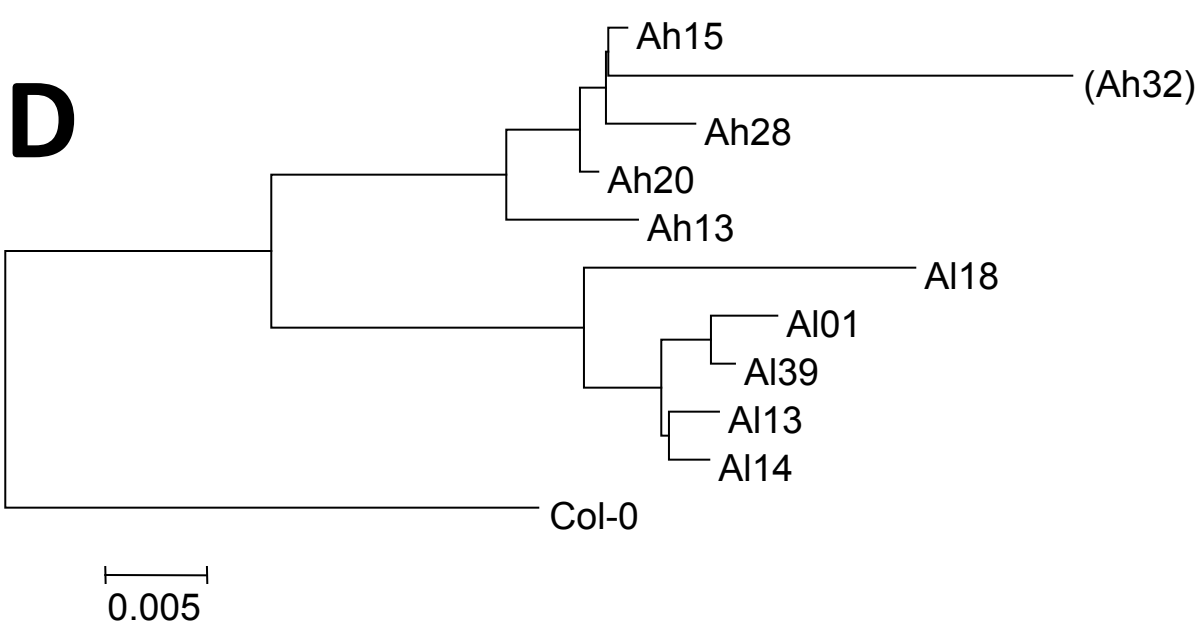

***At4g21340***

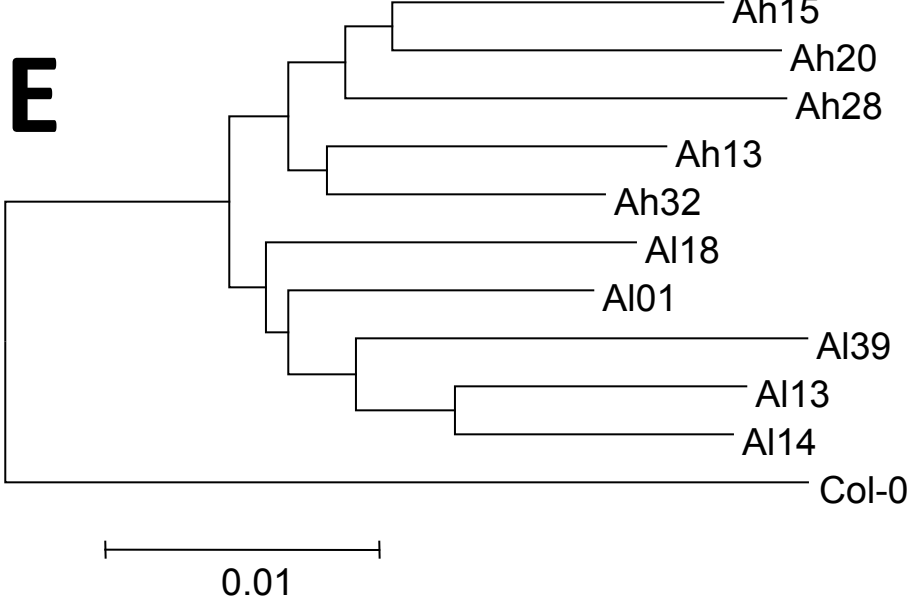

***U-box***

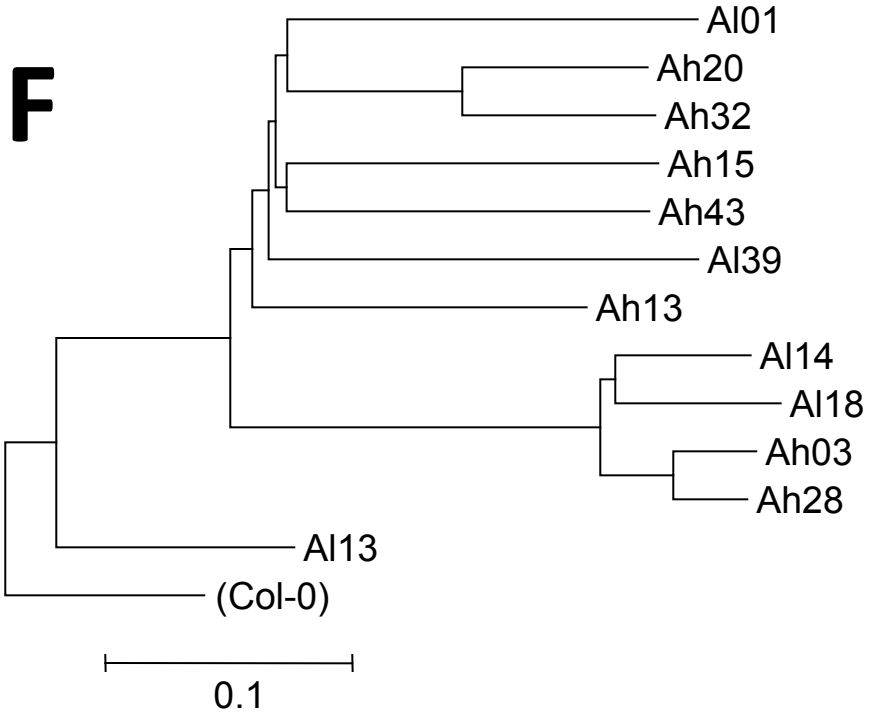

***SRK***

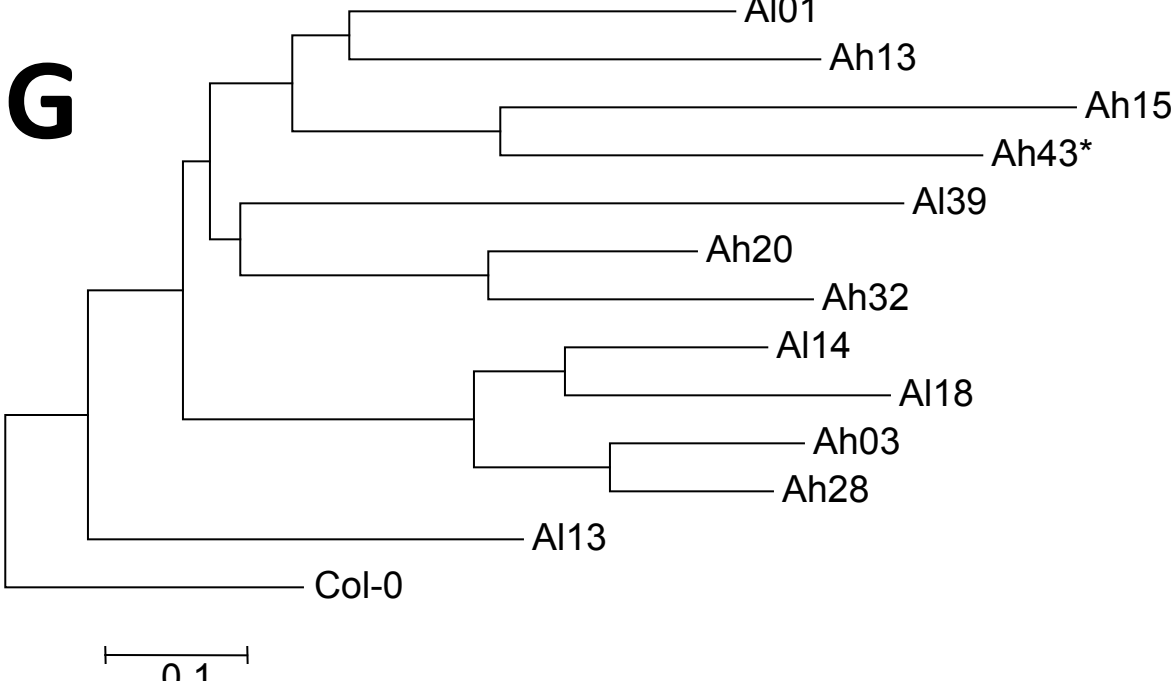

***SCR***

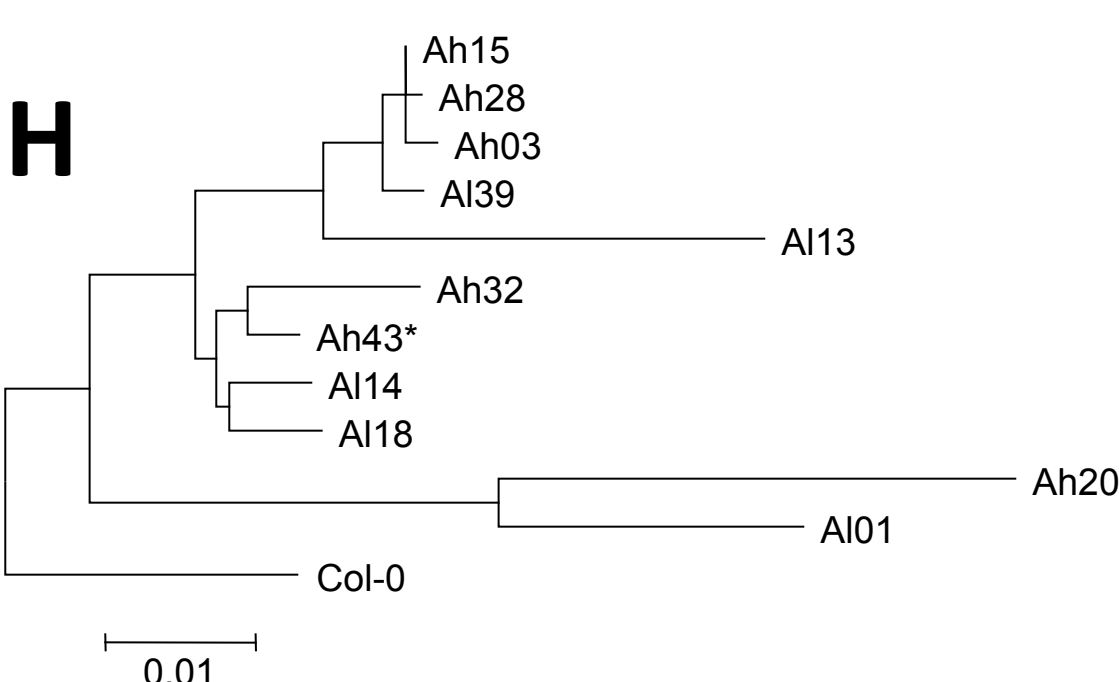

***ARK3***

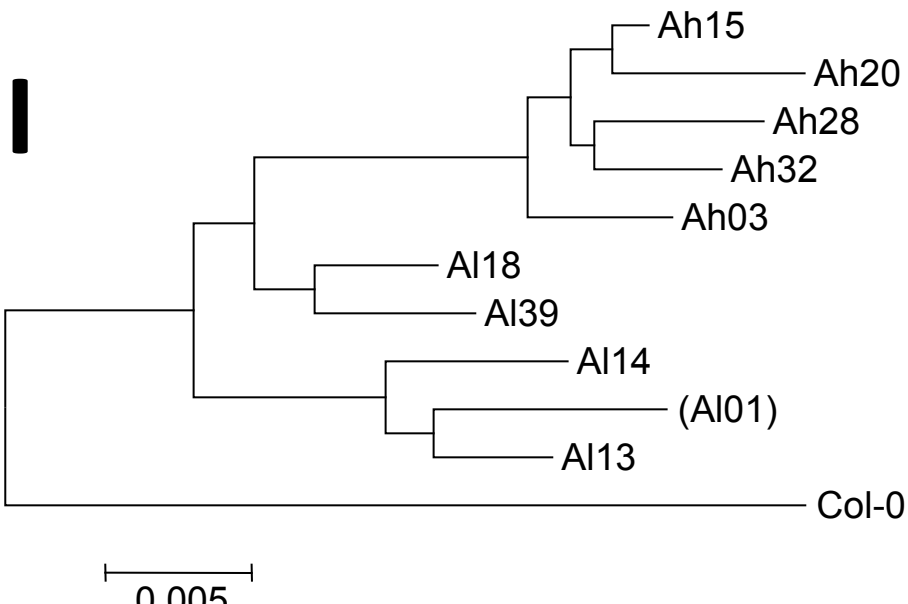

***At4g21390***

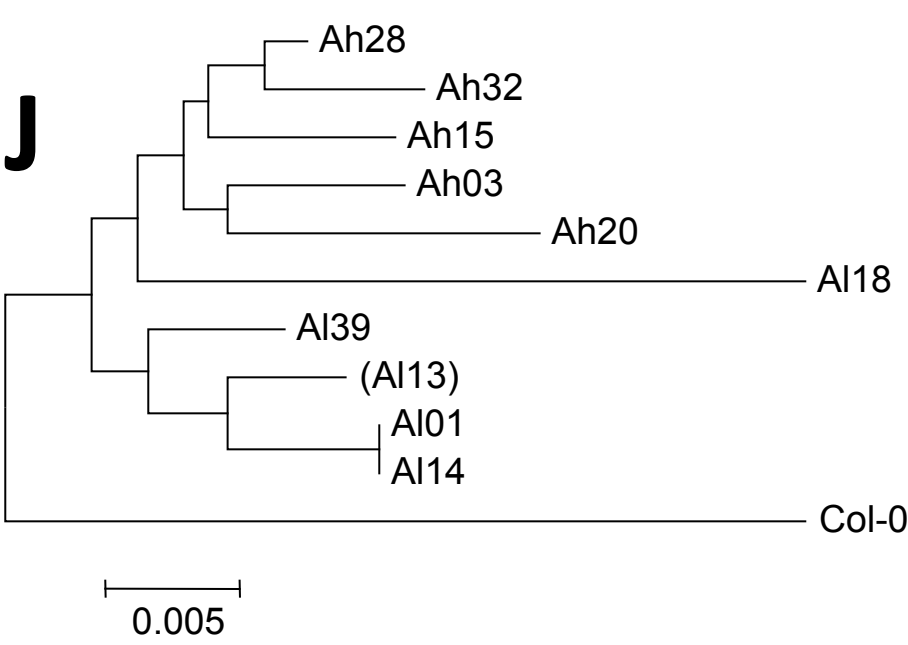

***At4g21400***

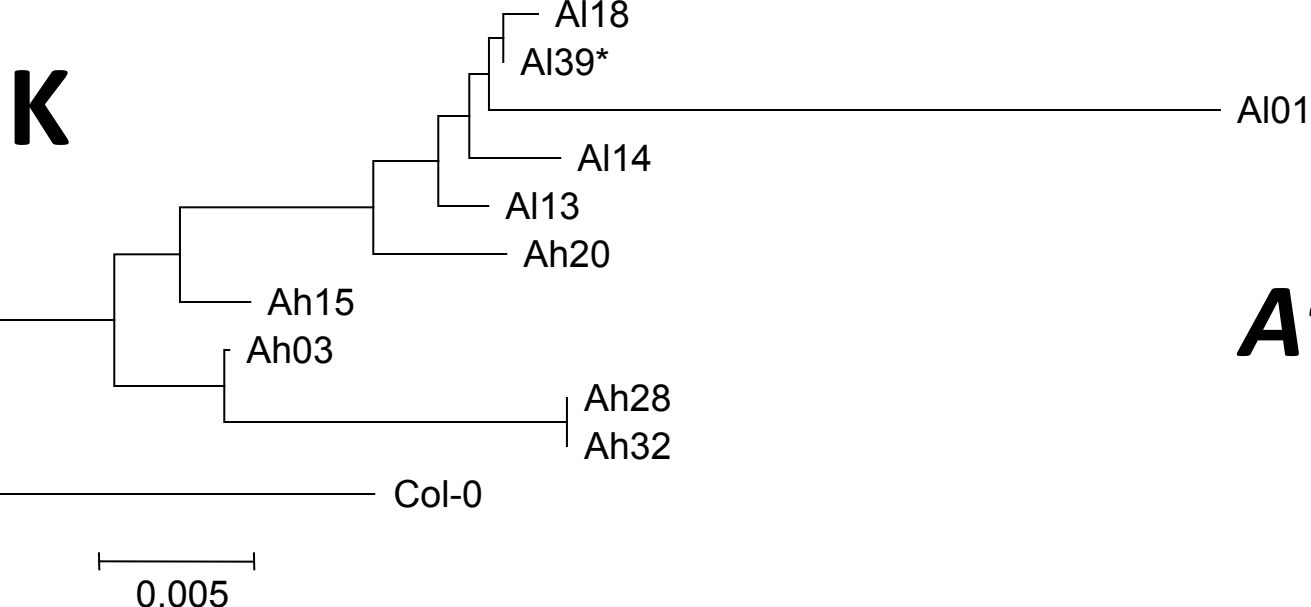

***At4g21410***

Supplement: Figure S4 — Separate phylogenies of the S-locus Region genes. Phylogenies were obtained by the Minimum Evolution method, and are based on protein sequences, with the A. thaliana reference sequences (Col-0) as outgroup. (PDF) [file pgen.1002495.s004.pdf]

Ah28

SRK

ARK3

Al01

Al14

Al18

Ah03

Al13

Al39

Ah13

Ah15

Ah20

Ah32

Ah43

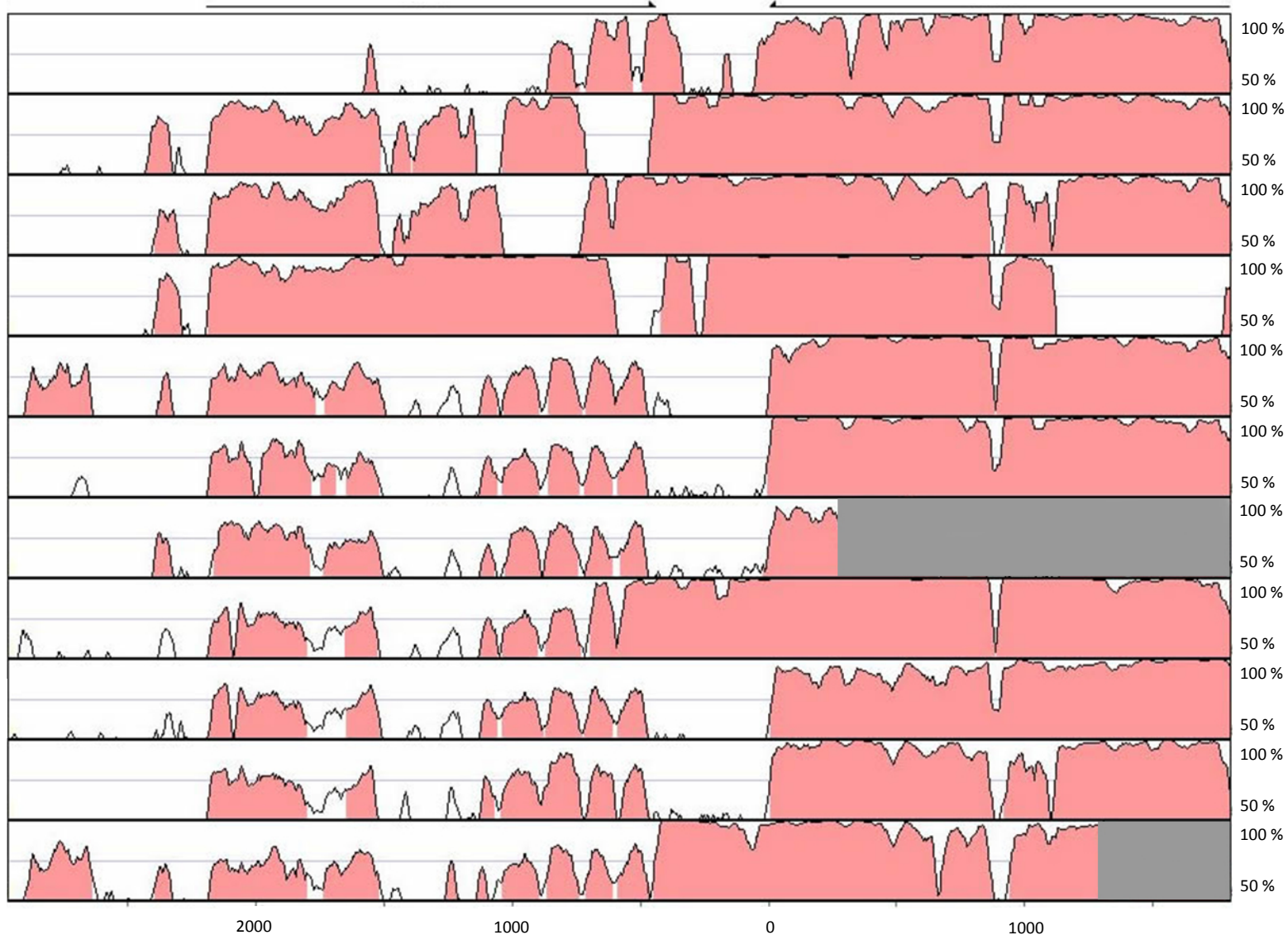

Supplement: Figure S5 — Sequence conservation in the SRK-ARK3 region between Ah28 (Class II) and each of the other haplotypes. Distance from homology breakpoint is indicated under the graph. (PDF) [file pgen.1002495.s005.pdf]

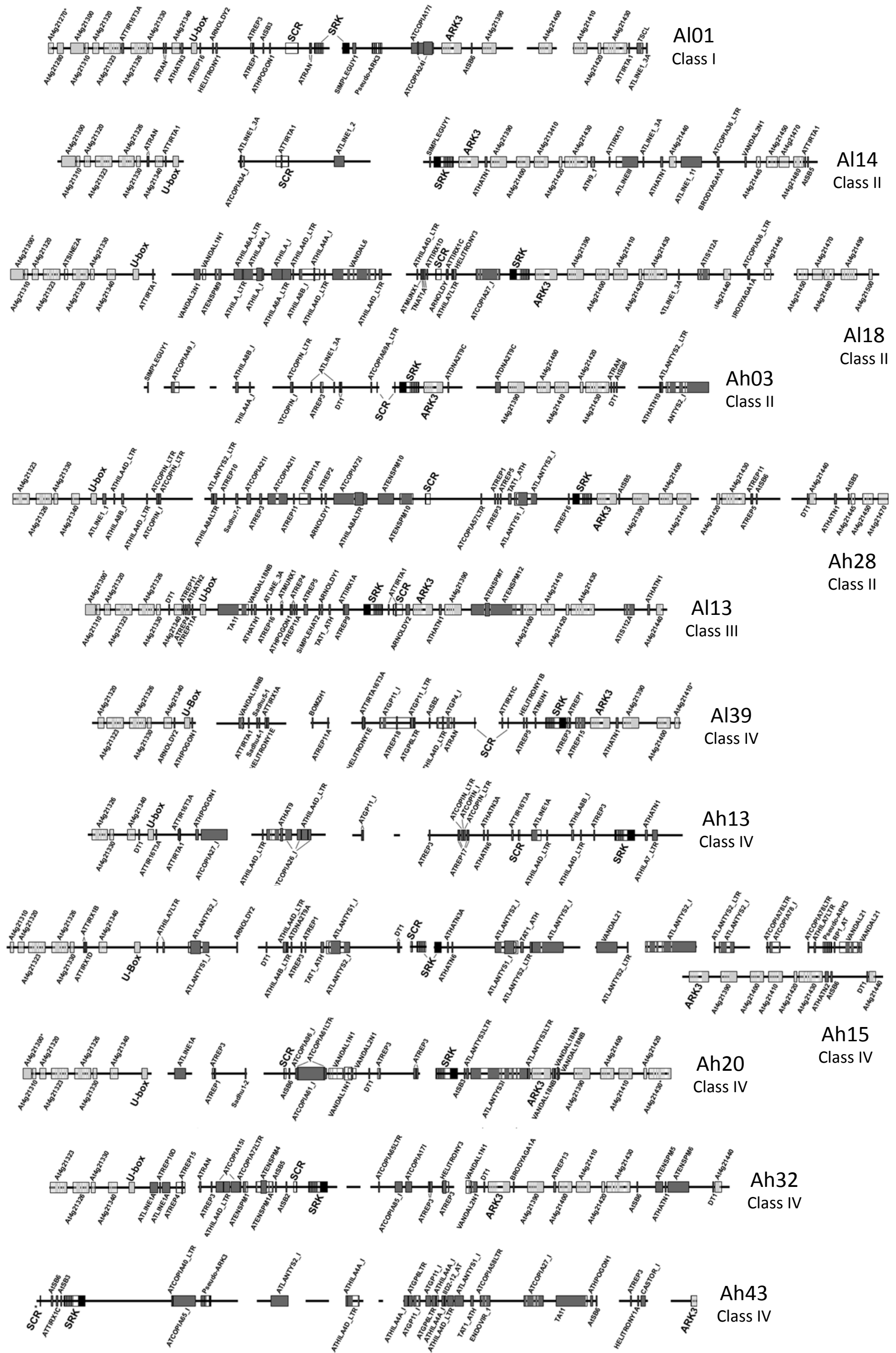

Supplement: Figure S6 — Annotation of genes and transposable elements for the 12 S-haplotypes. The S-locus genes are represented in black rectangles, with delimitation of their exons. Other genes are depicted in light gray. Transposable elements are shown in dark gray, and their fragmentation is indicated by white gaps. (PDF) [file pgen.1002495.s006.pdf]

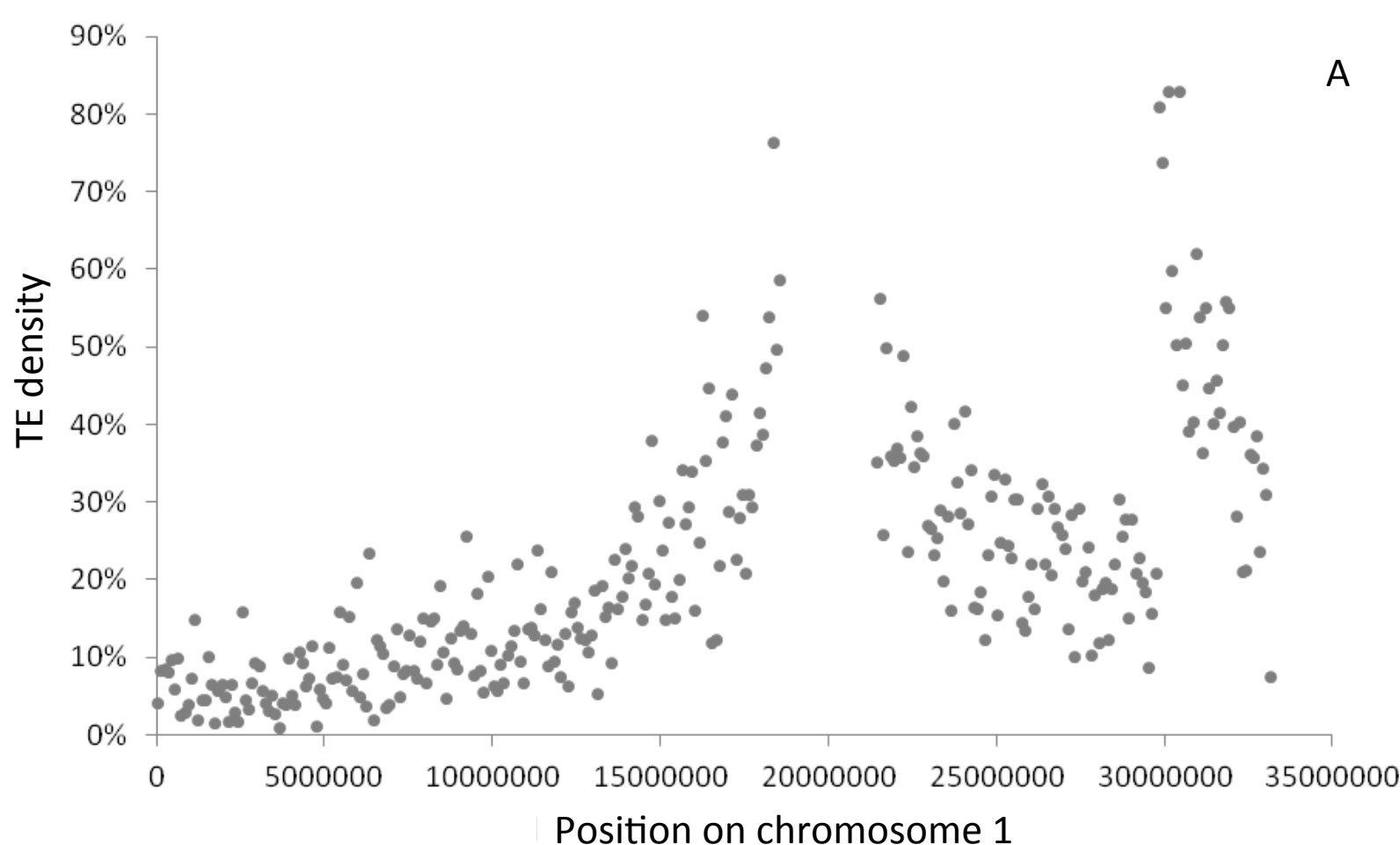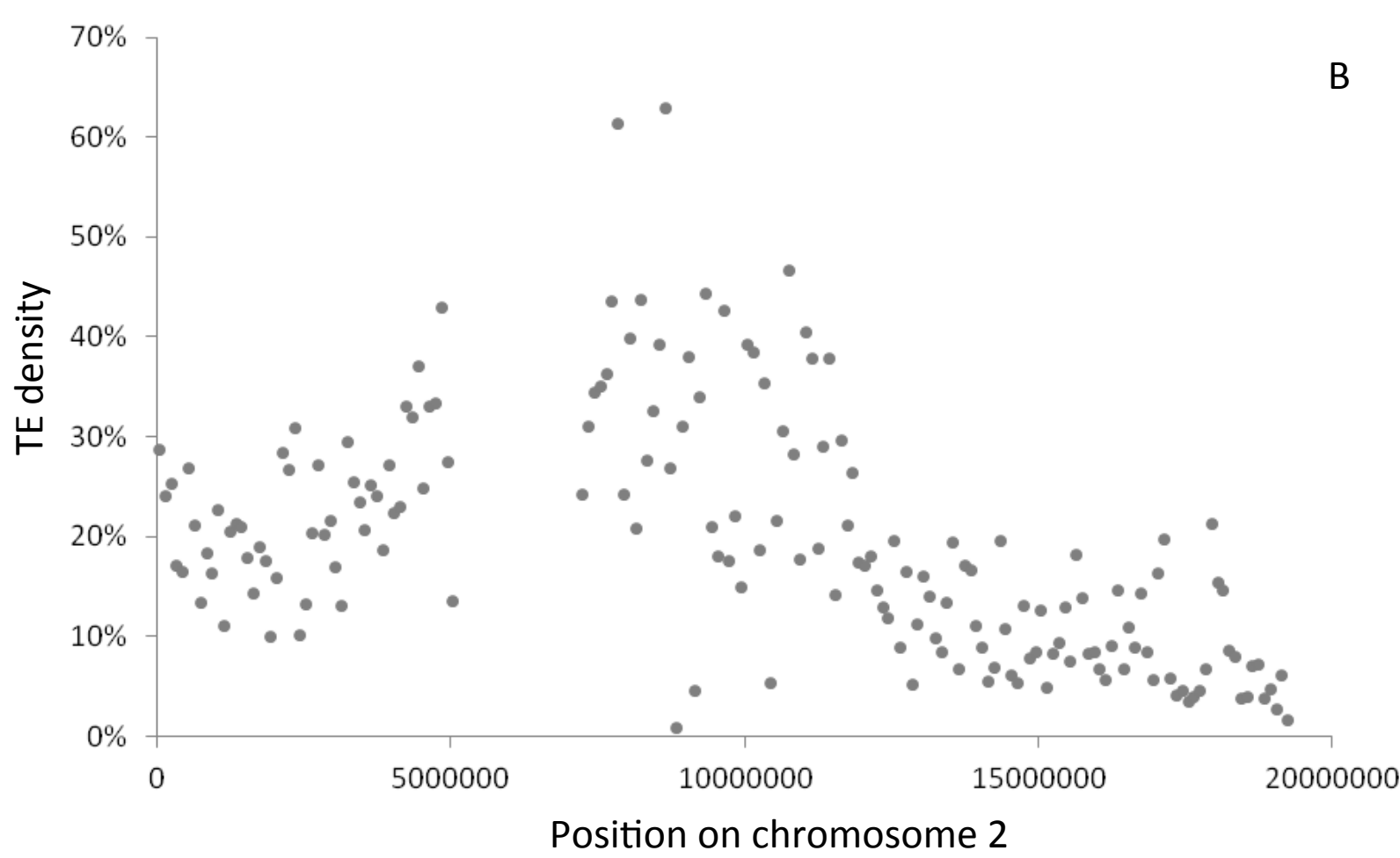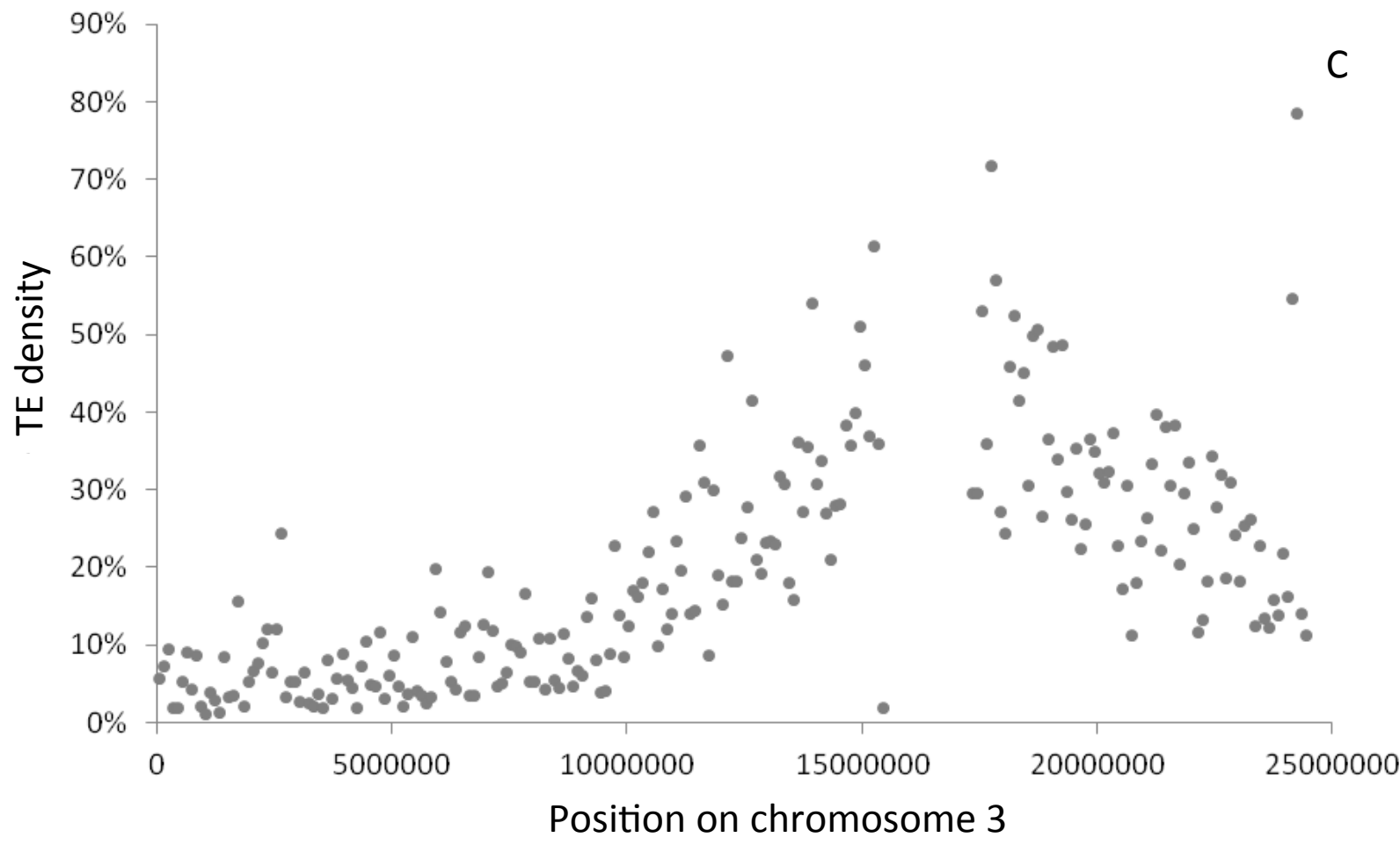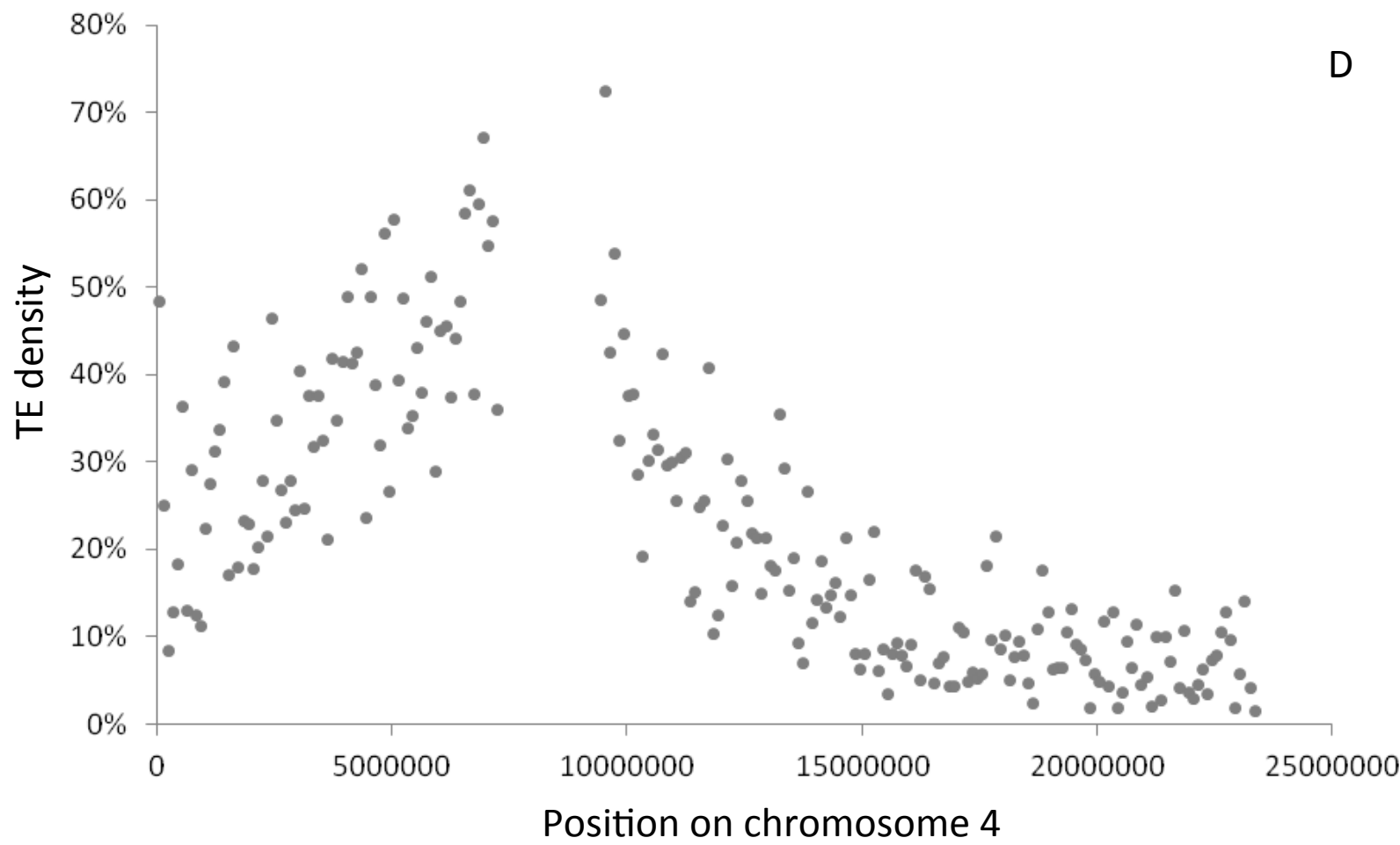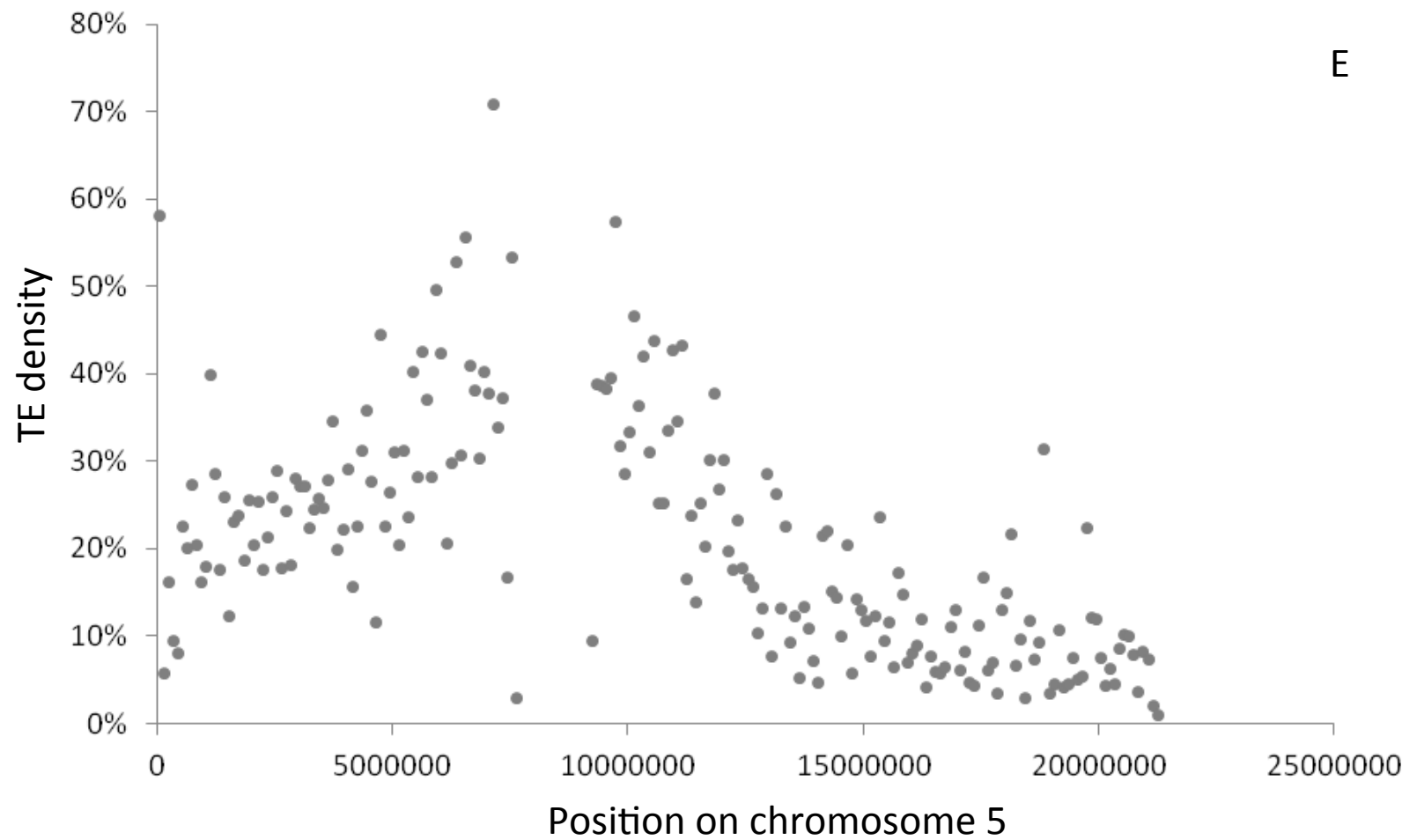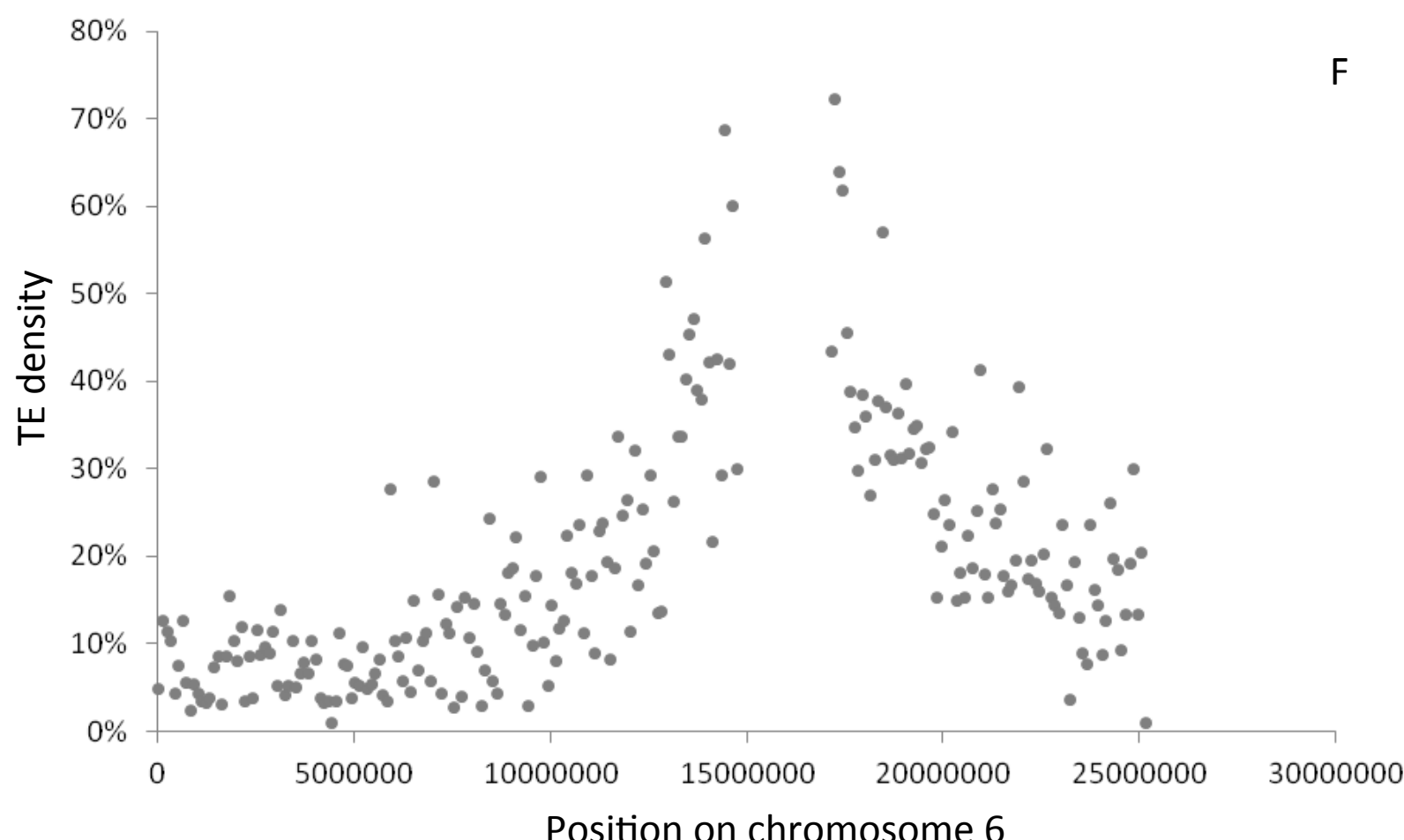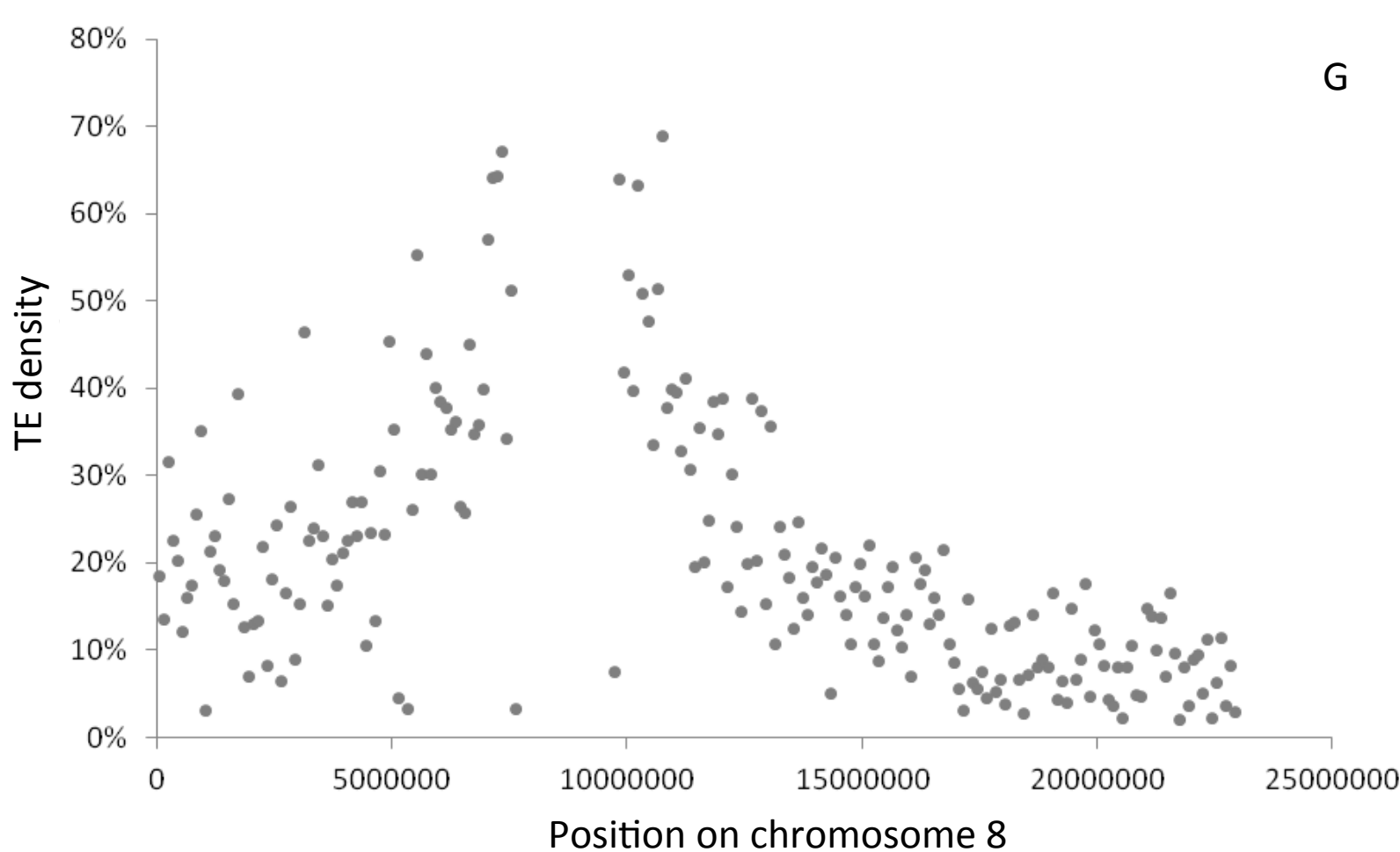

Supplement: Figure S7 — TE density along A. lyrata chromosomes 1 to 6 and chromosome 8. Transposable elements contents were calculated using CENSOR [72] for non overlapping windows of 100 kb. (PDF) [file pgen.1002495.s007.pdf]

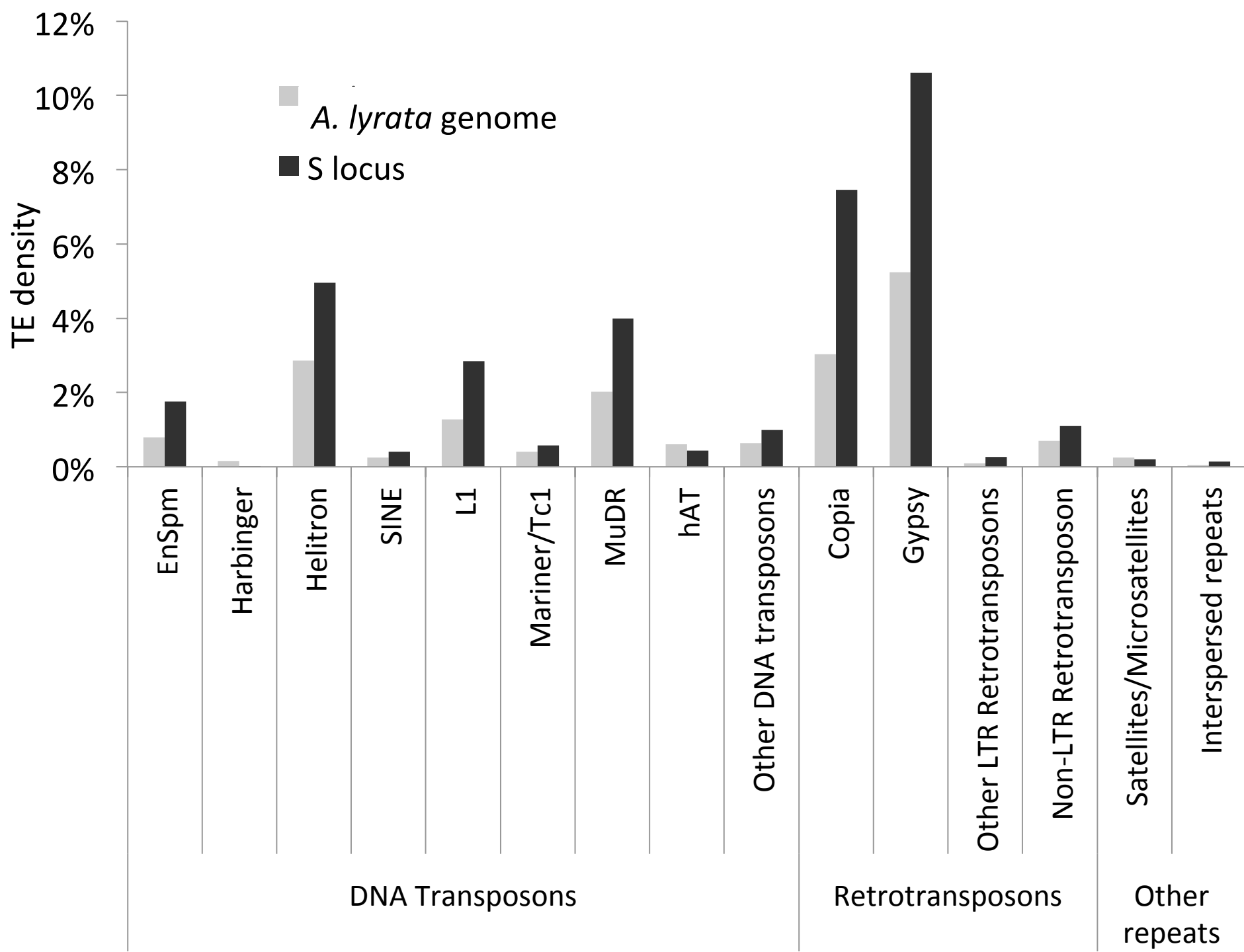

Supplement: Figure S8 — Comparative density in different families of transposable elements for the entire genome of A. lyrata, and the S-locus of A. lyrata and A. halleri. Transposable elements classification refers to Wicker et al. [104]. (PDF) [file pgen.1002495.s008.pdf]
