# Supplementary material for: Contrasted Patterns of Molecular Evolution in Dominant and Recessive Self-Incompatibility Haplotypes in Arabidopsis
Source: PLoS Genet. 2012 Mar 22;8(3):e1002495. doi: 10.1371/journal.pgen.1002495 (PMC3310759; doi:10.1371/journal.pgen.1002495)
Supplement: Table S1 — Description of the different clones. Two clones were necessary to cover the entire S-locus for three haplotypes : Al18, Ah28 and Ah15. (DOC) [file pgen.1002495.s009.doc]

**Supplementary table 1. Description of the different clones.
Two clones were necessary to cover the entire S-locus for three haplotypes : *Al18, Ah28* and *Ah15*.**

| **BAC** | **BAC size** | **Number of reads** | **Average size of reads** | **Coverage** | **Number of contigs** | **Genes at extremities** |
| --- | --- | --- | --- | --- | --- | --- |
|  |  |  |  |  |  |  |
| *Al01* | 100 937 | 10 900 | 331 | 35.74 | 4 | *At4g21270^a^ - At4g21430* |
| *Al14* | 117 539 | 54 361 | 344 | 159.09 | 3 | *At4g21300 - At4g21480* |
| *Al18* BAC1 | 88 062 | 15 066 | 372 | 63.64 | 3 | *At4g21300^a^ - SRK^a^* |
| *Al18* BAC2 | 96 061 | 5 126 | 346 | 18.46 | 4 | *SRK - At4g21500* |
| *Ah03* | 84 197 | 6 848 | 359 | 29.20 | 8 | *SCR - At4g21430* |
| *Ah28* BAC1 | 101 609 | 9 569 | 362 | 34.09 | 2 | *At4g21323 - SRK^a^* |
| *Ah28* BAC2 | 94 078 | 18 635 | 349 | 69.13 | 3 | *SCR - At4g21470* |
| *Al39* | 90 060 | 17 367 | 376 | 75.22 | 5 | *At4g21320 - At4g21410^a^* |
| *Ah13* | 88 292 | 11 931 | 348 | 47.03 | 5 | *At4g21326 - SRK* |
| *Ah15* BAC1 | 109 343 | 8 877 | 351 | 28.50 | 9 | *SCR - At4g21440* |
| *Ah15* BAC2 | 85 357 | 10 636 | 345 | 42.99 | 4 | *At4g21310 - SRK* |
| *Ah20* | 105 142 | 27 578 | 350 | 91.80 | 5 | *At4g21300^a^ - At4g21430^a^* |
| *Ah32* | 115 243 | 15 378 | 351 | 46.84 | 5 | *At4g21326 - At4g21440* |
| *Ah43* | 95 096 | 12 656 | 378 | 47.54 | 8 | *SCR^a^ - ARK3^a^* |
|  |  |  |  |  |  |  |

^a^ The sequence is incomplete because the BAC sequence ends into the gene
