# Supplementary material for: Contrasted Patterns of Molecular Evolution in Dominant and Recessive Self-Incompatibility Haplotypes in Arabidopsis
Source: PLoS Genet. 2012 Mar 22;8(3):e1002495. doi: 10.1371/journal.pgen.1002495 (PMC3310759; doi:10.1371/journal.pgen.1002495)
Supplement: Table S2 — List of the transposable elements detected in the BAC sequences. (DOC) [file pgen.1002495.s010.doc]

Supplementary table 2. List of the transposable elements detected in the BAC sequences.

| **Haplotype** | **Name** | **Type** | **Size** |
| --- | --- | --- | --- |
|  |  |  |  |
| *Al01* | ARNOLDY2 | MuDR | 156 |
| *Al01* | ATCOPIA17I | Copia | 2000 |
| *Al01* | ATCOPIA24I | Copia | 1924 |
| *Al01* | ATHATN3 | hAT | 457 |
| *Al01* | ATHPOGON1 | Mariner/Tc1 | 132 |
| *Al01* | ATLINE1_3A | L1 | 308 |
| *Al01* | ATRAN | DNA transposon | 116 |
| *Al01* | ATRAN | DNA transposon | 142 |
| *Al01* | ATRAN | DNA transposon | 148 |
| *Al01* | ATRAN | DNA transposon | 241 |
| *Al01* | ATREP1 | Helitron | 231 |
| *Al01* | ATREP16 | DNA transposon | 158 |
| *Al01* | ATREP3 | Helitron | 357 |
| *Al01* | AtSB3 | SINE | 249 |
| *Al01* | AtSB6 | SINE | 345 |
| *Al01* | ATTIR16T3A | Mariner/Tc1 | 491 |
| *Al01* | ATTIRTA1 | Mariner/Tc1 | 243 |
| *Al01* | HELITRONY1B | Helitron | 366 |
| *Al01* | SIMPLEGUY1 | MSAT | 706 |
| *Al01* | TSCL | Non LTR retrotransposon | 1153 |
| *Al14* | ATCOPIA34_I | Copia | 457 |
| *Al14* | ATCOPIA36_LTR | Copia | 68 |
| *Al14* | ATHATN1 | hAT | 480 |
| *Al14* | ATHATN1 | hAT | 504 |
| *Al14* | ATLINE1_11 | Non LTR retrotransposon | 3849 |
| *Al14* | ATLINE1_2 | L1 | 1911 |
| *Al14* | ATLINE1_3A | L1 | 80 |
| *Al14* | ATLINE1_3A | L1 | 87 |
| *Al14* | ATLINEIII | Non LTR retrotransposon | 2939 |
| *Al14* | ATN9_1 | MuDR | 858 |
| *Al14* | ATRAN | DNA transposon | 142 |
| *Al14* | ATRAN | DNA transposon | 116 |
| *Al14* | AtSB5 | SINE | 198 |
| *Al14* | ATTIRTA1 | Mariner/Tc1 | 60 |
| *Al14* | ATTIRTA1 | Mariner/Tc1 | 246 |
| *Al14* | ATTIRTA1 | Mariner/Tc1 | 239 |
| *Al14* | ATTIRX1D | DNA transposon | 339 |
| *Al14* | BRODYAGA1A | DNA transposon | 116 |
| *Al14* | SIMPLEGUY1 | MSAT | 115 |
| *Al14* | VANDAL2N1 | MuDR | 438 |
| *Al18* | ARNOLDY2 | MuDR | 307 |
| *Al18* | ATCOPIA27_I | Copia | 4217 |
| *Al18* | ATCOPIA36_LTR | Copia | 68 |
| *Al18* | ATENSPM9 | EnSpm | 1008 |
| *Al18* | ATHILA_I | Gypsy | 1968 |
| *Al18* | ATHILA_I | Gypsy | 1141 |
| *Al18* | ATHILA_LTR | Gypsy | 162 |
| *Al18* | ATHILA4A_I | Gypsy | 306 |
| *Al18* | ATHILA4D_LTR | Gypsy | 197 |
| *Al18* | ATHILA4D_LTR | Gypsy | 564 |
| *Al18* | ATHILA4D_LTR | Gypsy | 212 |
| *Al18* | ATHILA4D_LTR | Gypsy | 212 |
| *Al18* | ATHILA6A_I | Gypsy | 2096 |
| *Al18* | ATHILA6A_LTR | Gypsy | 1731 |
| *Al18* | ATHILA6A_LTR | Gypsy | 1565 |
| *Al18* | ATHILA7LTR | Gypsy | 233 |
| *Al18* | ATHILA8B_I | Gypsy | 768 |
| *Al18* | ATIS112A | Harbinger | 1978 |
| *Al18* | ATLINE1_3A | L1 | 332 |
| *Al18* | ATMUNX1 | MuDR | 101 |
| *Al18* | ATSINE2A | SINE | 180 |
| *Al18* | ATTIRTA1 | Mariner/Tc1 | 1937 |
| *Al18* | ATTIRX1C | DNA transposon | 191 |
| *Al18* | ATTIRX1D | DNA transposon | 345 |
| *Al18* | BRODYAGA1A | DNA transposon | 116 |
| *Al18* | HELITRONY3 | Helitron | 619 |
| *Al18* | TNAT1A | DNA transposon | 381 |
| *Al18* | TNAT1A | DNA transposon | 77 |
| *Al18* | TNAT1A | DNA transposon | 73 |
| *Al18* | VANDAL1N1 | MuDR | 231 |
| *Al18* | VANDAL2N1 | MuDR | 1190 |
| *Al18* | VANDAL6 | MuDR | 5665 |
| *Ah03* | ATCOPIA49_I | Copia | 302 |
| *Ah03* | ATCOPIA69A_LTR | Copia | 784 |
| *Ah03* | ATCOPIN_I | LTR Retrotransposon | 2904 |
| *Ah03* | ATCOPIN_LTR | LTR Retrotransposon | 302 |
| *Ah03* | ATDNA2T9C | MuDR | 290 |
| *Ah03* | ATDNA2T9C | MuDR | 431 |
| *Ah03* | ATHATN10 | hAT | 171 |
| *Ah03* | ATHILA4A_I | Gypsy | 552 |
| *Ah03* | ATHILA8B_I | Gypsy | 1727 |
| *Ah03* | ATLANTYS2_I | Gypsy | 6427 |
| *Ah03* | ATLANTYS2_LTR | Gypsy | 198 |
| *Ah03* | ATLINE1_3A | L1 | 1937 |
| *Ah03* | ATLINE1_3A | L1 | 65 |
| *Ah03* | ATRAN | DNA transposon | 1671 |
| *Ah03* | ATREP3 | Helitron | 395 |
| *Ah03* | AtSB6 | SINE | 1326 |
| *Ah03* | DT1 | Mariner/Tc1 | 335 |
| *Ah03* | DT1 | Mariner/Tc1 | 239 |
| *Ah03* | DT1 | Mariner/Tc1 | 148 |
| *Ah03* | SIMPLEGUY1 | MSAT | 130 |
| *Ah28* | ARNOLDY1 | MuDR | 431 |
| *Ah28* | ATCOPIA15I | Copia | 1727 |
| *Ah28* | ATCOPIA21I | Copia | 3145 |
| *Ah28* | ATCOPIA21I | Copia | 752 |
| *Ah28* | ATCOPIA57LTR | Copia | 126 |
| *Ah28* | ATCOPIA72_I | Copia | 784 |
| *Ah28* | ATCOPIA72_I | Copia | 4496 |
| *Ah28* | ATCOPIN_I | LTR Retrotransposon | 111 |
| *Ah28* | ATCOPIN_LTR | LTR Retrotransposon | 364 |
| *Ah28* | ATCOPIN_LTR | LTR Retrotransposon | 357 |
| *Ah28* | ATENSPM10 | EnSpm | 2904 |
| *Ah28* | ATENSPM10 | EnSpm | 2304 |
| *Ah28* | ATENSPM10 | EnSpm | 2304 |
| *Ah28* | ATENSPM10 | EnSpm | 2904 |
| *Ah28* | ATHATN1 | hAT | 480 |
| *Ah28* | ATHILA4D_LTR | Gypsy | 222 |
| *Ah28* | ATHILA4D_LTR | Gypsy | 233 |
| *Ah28* | ATHILA8ALTR | Gypsy | 1550 |
| *Ah28* | ATHILA8ALTR | Gypsy | 1550 |
| *Ah28* | ATHILA8ALTR | Gypsy | 656 |
| *Ah28* | ATHILA8B_I | Gypsy | 540 |
| *Ah28* | ATLANTYS1_I | Gypsy | 3294 |
| *Ah28* | ATLANTYS2_LTR | Gypsy | 427 |
| *Ah28* | ATLINE1_1 | L1 | 635 |
| *Ah28* | ATREP1 | Helitron | 192 |
| *Ah28* | ATREP10 | Helitron | 142 |
| *Ah28* | ATREP11 | Helitron | 115 |
| *Ah28* | ATREP11 | Helitron | 207 |
| *Ah28* | ATREP11A | Helitron | 607 |
| *Ah28* | ATREP16 | DNA transposon | 241 |
| *Ah28* | ATREP2 | Helitron | 268 |
| *Ah28* | ATREP3 | Helitron | 335 |
| *Ah28* | ATREP3 | Helitron | 462 |
| *Ah28* | ATREP5 | Helitron | 280 |
| *Ah28* | ATREP5 | Helitron | 285 |
| *Ah28* | AtSB3 | SINE | 301 |
| *Ah28* | AtSB5 | SINE | 148 |
| *Ah28* | AtSB6 | SINE | 335 |
| *Ah28* | DT1 | Mariner/Tc1 | 239 |
| *Ah28* | Sadhu7-1 | SINE | 484 |
| *Ah28* | TAT1_ATH | Gypsy | 613 |
| *Al13* | ARNOLDY1 | MuDR | 443 |
| *Al13* | ARNOLDY2 | MuDR | 845 |
| *Al13* | ATENSPM12 | EnSpm | 7343 |
| *Al13* | ATENSPM7 | EnSpm | 878 |
| *Al13* | ATHATN1 | hAT | 514 |
| *Al13* | ATHATN1 | hAT | 472 |
| *Al13* | ATHATN1 | hAT | 498 |
| *Al13* | ATHATN2 | hAT | 569 |
| *Al13* | ATHPOGON1 | Mariner/Tc1 | 83 |
| *Al13* | ATIS112A | Harbinger | 1774 |
| *Al13* | ATLINE1_3A | L1 | 49 |
| *Al13* | ATMUNX1 | MuDR | 81 |
| *Al13* | ATMUNX1 | MuDR | 228 |
| *Al13* | ATREP11 | Helitron | 168 |
| *Al13* | ATREP11A | Helitron | 180 |
| *Al13* | ATREP11A | Helitron | 194 |
| *Al13* | ATREP16 | DNA transposon | 183 |
| *Al13* | ATREP4 | Helitron | 232 |
| *Al13* | ATREP4 | Helitron | 370 |
| *Al13* | ATREP4 | Helitron | 247 |
| *Al13* | ATREP5 | Helitron | 888 |
| *Al13* | ATREP9 | Helitron | 912 |
| *Al13* | ATTIRTA1 | Mariner/Tc1 | 109 |
| *Al13* | ATTIRTA1 | Mariner/Tc1 | 252 |
| *Al13* | ATTIRX1A | DNA transposon | 330 |
| *Al13* | DT1 | Mariner/Tc1 | 269 |
| *Al13* | SIMPLEHAT2 | MSAT | 247 |
| *Al13* | TA11 | L2 | 4782 |
| *Al13* | TAT1_ATH | Gypsy | 423 |
| *Al13* | VANDAL18NB | MuDR | 263 |
| *Al39* | ARNOLDY2 | MuDR | 156 |
| *Al39* | ATGP11_I | Gypsy | 1044 |
| *Al39* | ATGP11_LTR | Gypsy | 634 |
| *Al39* | ATGP4_I | Gypsy | 1134 |
| *Al39* | ATGP6LTR | Gypsy | 268 |
| *Al39* | ATHATN1 | hAT | 462 |
| *Al39* | ATHILA4D_LTR | Gypsy | 204 |
| *Al39* | ATHPOGON1 | Mariner/Tc1 | 100 |
| *Al39* | ATMUN1 | MuDR | 135 |
| *Al39* | ATRAN | DNA transposon | 61 |
| *Al39* | ATREP1 | Helitron | 232 |
| *Al39* | ATREP11A | Helitron | 211 |
| *Al39* | ATREP15 | Helitron | 1571 |
| *Al39* | ATREP18 | Interspersed repeat | 183 |
| *Al39* | ATREP3 | Helitron | 351 |
| *Al39* | ATREP5 | Helitron | 239 |
| *Al39* | AtSB2 | SINE | 170 |
| *Al39* | ATTIR16T3A | Mariner/Tc1 | 485 |
| *Al39* | ATTIRTA1 | Mariner/Tc1 | 244 |
| *Al39* | ATTIRX1A | DNA transposon | 368 |
| *Al39* | ATTIRX1C | DNA transposon | 309 |
| *Al39* | BOMZH1 | MuDR | 286 |
| *Al39* | HELITRONY1B | Helitron | 139 |
| *Al39* | HELITRONY1E | Helitron | 445 |
| *Al39* | HELITRONY1E | Helitron | 200 |
| *Al39* | Sadhu4-1 | SINE | 113 |
| *Al39* | Sadhu5-1 | SINE | 347 |
| *Al39* | VANDAL18NB | MuDR | 602 |
| *Al39* | VANDAL18NB | MuDR | 108 |
| *Ah13* | ATCOPIA26I | Copia | 544 |
| *Ah13* | ATCOPIA26I | Copia | 828 |
| *Ah13* | ATCOPIA26I | Copia | 2807 |
| *Ah13* | ATCOPIA27_I | Copia | 4797 |
| *Ah13* | ATCOPIN_I | LTR Retrotransposon | 110 |
| *Ah13* | ATCOPIN_LTR | LTR Retrotransposon | 357 |
| *Ah13* | ATCOPIN_LTR | LTR Retrotransposon | 356 |
| *Ah13* | ATGP11_I | Gypsy | 355 |
| *Ah13* | ATHAT9 | hAT | 491 |
| *Ah13* | ATHATN1 | hAT | 473 |
| *Ah13* | ATHATN3A | hAT | 160 |
| *Ah13* | ATHATN6 | hAT | 211 |
| *Ah13* | ATHATN6 | hAT | 94 |
| *Ah13* | ATHILA4_LTR | Gypsy | 307 |
| *Ah13* | ATHILA4D_LTR | Gypsy | 151 |
| *Ah13* | ATHILA4D_LTR | Gypsy | 153 |
| *Ah13* | ATHILA4D_LTR | Gypsy | 1198 |
| *Ah13* | ATHILA7LTR | Gypsy | 966 |
| *Ah13* | ATHILA8B_I | Gypsy | 514 |
| *Ah13* | ATHPOGON1 | Mariner/Tc1 | 567 |
| *Ah13* | ATLINE1A | Non LTR retrotransposon | 1141 |
| *Ah13* | ATREP17 | DNA transposon | 233 |
| *Ah13* | ATREP17 | DNA transposon | 188 |
| *Ah13* | ATREP17 | DNA transposon | 192 |
| *Ah13* | ATREP17 | DNA transposon | 233 |
| *Ah13* | ATREP3 | Helitron | 242 |
| *Ah13* | ATREP3 | Helitron | 315 |
| *Ah13* | ATTIR16T3A | Mariner/Tc1 | 433 |
| *Ah13* | ATTIR16T3A | Mariner/Tc1 | 252 |
| *Ah13* | ATTIR16T3A | Mariner/Tc1 | 176 |
| *Ah13* | ATTIRTA1 | Mariner/Tc1 | 262 |
| *Ah13* | DT1 | Mariner/Tc1 | 270 |
| *Ah15* | ARNOLDY2 | MuDR | 185 |
| *Ah15* | ATCOPIA78_I | Copia | 920 |
| *Ah15* | ATCOPIA78LTR | Copia | 302 |
| *Ah15* | ATCOPIA78LTR | Copia | 133 |
| *Ah15* | ATDNA2T9A | MuDR | 179 |
| *Ah15* | ATHATN2 | hAT | 655 |
| *Ah15* | ATHATN3A | hAT | 160 |
| *Ah15* | ATHATN6 | hAT | 122 |
| *Ah15* | ATHILA4B_LTR | Gypsy | 147 |
| *Ah15* | ATHILA4D_LTR | Gypsy | 748 |
| *Ah15* | ATHILA7LTR | Gypsy | 328 |
| *Ah15* | ATHILA7LTR | Gypsy | 378 |
| *Ah15* | ATHILA7LTR | Gypsy | 353 |
| *Ah15* | ATLANTYS1_I | Gypsy | 1902 |
| *Ah15* | ATLANTYS1_I | Gypsy | 1903 |
| *Ah15* | ATLANTYS1_I | Gypsy | 1820 |
| *Ah15* | ATLANTYS2_I | Gypsy | 1345 |
| *Ah15* | ATLANTYS2_I | Gypsy | 1153 |
| *Ah15* | ATLANTYS2_I | Gypsy | 1333 |
| *Ah15* | ATLANTYS2_I | Gypsy | 2135 |
| *Ah15* | ATLANTYS2_I | Gypsy | 1317 |
| *Ah15* | ATLANTYS2_I | Gypsy | 5540 |
| *Ah15* | ATLANTYS2_I | Gypsy | 7653 |
| *Ah15* | ATLANTYS2_I | Gypsy | 2135 |
| *Ah15* | ATLANTYS2_LTR | Gypsy | 333 |
| *Ah15* | ATLANTYS2_LTR | Gypsy | 440 |
| *Ah15* | ATLANTYS2_LTR | Gypsy | 88 |
| *Ah15* | ATLANTYS2_LTR | Gypsy | 333 |
| *Ah15* | ATREP1 | Helitron | 194 |
| *Ah15* | ATREP3 | Helitron | 312 |
| *Ah15* | AtSB6 | SINE | 350 |
| *Ah15* | ATTIRX1B | DNA transposon | 139 |
| *Ah15* | ATTIRX1B | DNA transposon | 175 |
| *Ah15* | ATTIRX1D | DNA transposon | 339 |
| *Ah15* | DT1 | Mariner/Tc1 | 121 |
| *Ah15* | DT1 | Mariner/Tc1 | 66 |
| *Ah15* | DT1 | Mariner/Tc1 | 214 |
| *Ah15* | DT1 | Mariner/Tc1 | 239 |
| *Ah15* | RP1_AT | DNA transposon | 125 |
| *Ah15* | TAT1_ATH | Gypsy | 613 |
| *Ah15* | TAT1_ATH | Gypsy | 613 |
| *Ah15* | VANDAL21 | MuDR | 3815 |
| *Ah15* | VANDAL21 | MuDR | 2311 |
| *Ah20* | ATCOPIA61_I | Copia | 4665 |
| *Ah20* | ATCOPIA61LTR | Copia | 172 |
| *Ah20* | ATCOPIA61LTR | Copia | 178 |
| *Ah20* | ATCOPIA96_I | Copia | 710 |
| *Ah20* | ATLANTYS3I | Gypsy | 7890 |
| *Ah20* | ATLANTYS3LTR | Gypsy | 493 |
| *Ah20* | ATLANTYS3LTR | Gypsy | 501 |
| *Ah20* | ATLINE1A | Non LTR retrotransposon | 2138 |
| *Ah20* | ATREP1 | Helitron | 228 |
| *Ah20* | ATREP3 | Helitron | 389 |
| *Ah20* | ATREP3 | Helitron | 522 |
| *Ah20* | ATREP3 | Helitron | 579 |
| *Ah20* | ATREP3 | Helitron | 350 |
| *Ah20* | AtSB3 | SINE | 224 |
| *Ah20* | AtSB6 | SINE | 344 |
| *Ah20* | DT1 | Mariner/Tc1 | 104 |
| *Ah20* | Sadhu1-2 | SINE | 241 |
| *Ah20* | VANDAL18NA | MuDR | 300 |
| *Ah20* | VANDAL18NB | MuDR | 423 |
| *Ah20* | VANDAL18NB | MuDR | 528 |
| *Ah20* | VANDAL1N1 | MuDR | 539 |
| *Ah20* | VANDAL1N1 | MuDR | 807 |
| *Ah20* | VANDAL2N1 | MuDR | 455 |
| *Ah32* | ATCOPIA15I | Copia | 1422 |
| *Ah32* | ATCOPIA15I | Copia | 1700 |
| *Ah32* | ATCOPIA17I | Copia | 1247 |
| *Ah32* | ATCOPIA65LTR | Copia | 252 |
| *Ah32* | ATCOPIA72LTR | Copia | 75 |
| *Ah32* | ATCOPIA85_I | Copia | 1376 |
| *Ah32* | ATENSPM1 | EnSpm | 640 |
| *Ah32* | ATENSPM1A | EnSpm | 716 |
| *Ah32* | ATENSPM4 | EnSpm | 1150 |
| *Ah32* | ATENSPM5 | EnSpm | 1354 |
| *Ah32* | ATENSPM6 | EnSpm | 3909 |
| *Ah32* | ATHATN1 | hAT | 279 |
| *Ah32* | ATHILA4D_LTR | Gypsy | 1191 |
| *Ah32* | ATLINE1A | Non LTR retrotransposon | 1465 |
| *Ah32* | ATLINE1A | Non LTR retrotransposon | 1504 |
| *Ah32* | ATRAN | DNA transposon | 366 |
| *Ah32* | ATREP10D | Helitron | 109 |
| *Ah32* | ATREP13 | Helitron | 527 |
| *Ah32* | ATREP15 | Helitron | 178 |
| *Ah32* | ATREP3 | Helitron | 262 |
| *Ah32* | ATREP3 | Helitron | 340 |
| *Ah32* | ATREP3 | Helitron | 494 |
| *Ah32* | ATREP3 | Helitron | 295 |
| *Ah32* | ATREP4 | Helitron | 291 |
| *Ah32* | AtSB2 | SINE | 97 |
| *Ah32* | AtSB5 | SINE | 99 |
| *Ah32* | AtSB6 | SINE | 336 |
| *Ah32* | BRODYAGA1A | DNA transposon | 115 |
| *Ah32* | DT1 | Mariner/Tc1 | 236 |
| *Ah32* | DT1 | Mariner/Tc1 | 143 |
| *Ah32* | HELITRONY3 | Helitron | 661 |
| *Ah32* | VANDAL1N1 | MuDR | 268 |
| *Ah32* | VANDAL2N1 | MuDR | 1228 |
| *Ah43* | ATCOPIA27_I | Copia | 3399 |
| *Ah43* | ATCOPIA40_LTR | Copia | 181 |
| *Ah43* | ATCOPIA58LTR | Copia | 312 |
| *Ah43* | ATCOPIA65_I | Copia | 4093 |
| *Ah43* | ATGP11_I | Gypsy | 943 |
| *Ah43* | ATGP11_I | Gypsy | 953 |
| *Ah43* | ATGP6LTR | Gypsy | 819 |
| *Ah43* | ATGP6LTR | Gypsy | 819 |
| *Ah43* | ATHILA4A_I | Gypsy | 363 |
| *Ah43* | ATHILA4A_I | Gypsy | 830 |
| *Ah43* | ATHILA4A_I | Gypsy | 369 |
| *Ah43* | ATHILA4A_I | Gypsy | 525 |
| *Ah43* | ATHILA4D_LTR | Gypsy | 1195 |
| *Ah43* | ATHILA4D_LTR | Gypsy | 1175 |
| *Ah43* | ATHPOGON1 | Mariner/Tc1 | 551 |
| *Ah43* | ATLANTYS1_I | Gypsy | 1704 |
| *Ah43* | ATLANTYS2_I | Gypsy | 3206 |
| *Ah43* | ATREP3 | Helitron | 281 |
| *Ah43* | AtSB3 | SINE | 278 |
| *Ah43* | AtSB6 | SINE | 347 |
| *Ah43* | AtSB6 | SINE | 345 |
| *Ah43* | ATTIRX1C | DNA transposon | 394 |
| *Ah43* | CASTOR_I | Copia | 980 |
| *Ah43* | ENDOVIR1_I | Copia | 1585 |
| *Ah43* | HELITRONY1A | Helitron | 154 |
| *Ah43* | IID2-12_AT | Interspersed repeat | 396 |
| *Ah43* | TA11 | L1 | 5452 |
| *Ah43* | TAT1_ATH | Gypsy | 613 |
|  |  |  |  |
