# Supplementary material for: Contrasted Patterns of Molecular Evolution in Dominant and Recessive Self-Incompatibility Haplotypes in Arabidopsis
Source: PLoS Genet. 2012 Mar 22;8(3):e1002495. doi: 10.1371/journal.pgen.1002495 (PMC3310759; doi:10.1371/journal.pgen.1002495)
Supplement: Table S3 — Primers pairs used to validate BAC clones by PCR amplification. These primers were defined to amplify SRK (primer Sh04), U-box (primer B80) and ARK3 (primer ARK3) genes. (DOC) [file pgen.1002495.s011.doc]

**Supplementary table 3. Primers pairs used to validate BAC clones by PCR amplification.** These primers were defined to amplify *SRK* (primer Sh04), *U-box* (primer B80) and *ARK3* (primer ARK3) genes.

| **Name** | **Forward primer** | **Reverse primer** | **Product size** |
| --- | --- | --- | --- |
|  |  |  |  |
| Sh04 | ACGCCGTACAGTTACAATGT | GACAATAAATACCGTAGACG | 373 |
| B80 | TGGRTCRTATCCTGATGCAA | TCCGCTACAACAACACAAGC | 348 |
| ARK3 | TTGTGCGGTTGAAGAAGATG | GGAGAAGGAACTAACCGAKA | 255 |
|  |  |  |  |
